# Novel transcriptional responses to heat revealed by turning up the heat at night

**DOI:** 10.1007/s11103-019-00873-3

**Published:** 2019-05-06

**Authors:** Dmitry O. Grinevich, Jigar S. Desai, Kevin P. Stroup, Jiaqi Duan, Erin Slabaugh, Colleen J. Doherty

**Affiliations:** Department of Molecular and Structural Biochemistry, North Carolina State University, Raleigh, USA

**Keywords:** Heat shock, Diel regulation, Transcriptional networks, Abiotic stress, Gating of plant responses, Circadian clock

## Abstract

**Key message:**

The circadian clock controls many molecular activities, impacting experimental interpretation. We quantify the genome-wide effects of time-of-day on the heat-shock response and the effects of “diurnal bias” in stress experiments.

**Abstract:**

Heat stress has significant adverse effects on plant productivity worldwide. Most experiments examining heat stress are performed during daytime hours, generating a ‘diurnal bias’ in the pathways and regulatory mechanisms identified. Such bias may confound downstream interpretations and limit our understanding of the full response to heat stress. Here we show that the transcriptional and physiological responses to a sudden heat shock in *Arabidopsis* are profoundly sensitive to the time of day. We observe that plant tolerance and acclimation to heat shock vary throughout the day and are maximal at dusk. Consistently, over 75% of heat-responsive transcripts show a time of day-dependent response, including many previously characterized heat-response genes. This temporal sensitivity implies a complex interaction between time and temperature where daily variations in basal transcription influence thermotolerance. When we examined these transcriptional responses, we uncovered novel night-response genes and *cis*-regulatory elements, underpinning new aspects of heat stress responses not previously appreciated. Exploiting this temporal variation can be applied to most environmental responses to understand the underlying network wiring. Therefore, we propose that using time as a perturbagen is an approach that will enhance our understanding of plant regulatory networks and responses to environmental stresses.

**Electronic supplementary material:**

The online version of this article (10.1007/s11103-019-00873-3) contains supplementary material, which is available to authorized users.

## Background

The productivity of crops suffers from periods of increased temperatures (Siebers et al. 2015; Lesk et al. 2016). Crop losses from heat and drought events have cost an estimated 237 billion dollars USD globally in the last 55 years, resulting in imminent threats to global food security (Mehrabi and Ramankutty 2017). As the number of extreme high-temperature events continues to increase (Powell and Reinhard 2015), the negative impact on crops is expected to rise (Stocker et al. 2013; Muller and Robertson 2014). In the face of adverse climate conditions, there is a need for improved breeding programs to sustain agricultural yield (Lobell and Gourdji 2012), and a full understanding of heat stress responses is key to achieving this goal (Mickelbart et al. 2015; Driedonks et al. 2016).

Both exogenous and endogenous factors influence how plants respond to a heat event. Exogenously, the duration, intensity, and rate of temperature increase may influence heat responses and survival in plants (Wahid et al. 2007). Examples of endogenous factors include genotype differences, developmental state, and the inherent ability to acclimate (Wahid et al. 2007; Zinn et al. 2010; Bac-Molenaar et al. 2015; Xu et al. 2017). Previous exposure to a heat event can also improve the response to subsequent heat events through acquired thermotolerance, also known as heat stress memory (Alexandrov et al. 1961; Key et al. 1981; Nover et al. 1983; Vierling 1991; Howarth and Ougham 1993; Larkindale and Knight 2002; Larkindale and Vierling 2007; Rosenthal et al. 2014). Plants utilize three primary mechanisms to deal with heat stress: avoidance, tolerance, and escape. Heat avoidance includes leaf rolling or bud abscission (Ascough et al. 2005; Hasanuzzaman et al. 2013), while tolerance involves altering the plants’ physiology, such as the accumulation of osmoprotectants and anti-oxidants, to maintain productivity in high temperatures. Finally, heat escape mechanisms enable a plant to maintain productivity in high-temperature conditions through the phenological regulation of activities. Temperature sensitive activities are restricted to cooler nighttime temperatures, and the pace of developmental processes can be altered to avoid the hottest part of the season. However, unpredictable changes in the timing of weather conditions, e.g., sporadic heat waves and warmer nighttime temperatures, could reduce the effectiveness of heat escape strategies (Cannell et al. 1986; Peng et al. 2004; Schwartz et al. 2006; Welch et al. 2010; Mohammed and Tarpley 2011; Lyman et al. 2013; Coast et al. 2015; Guo et al. 2015; Jagadish et al. 2015; Houston et al. 2017; Aiqing et al. 2018).

The ability to anticipate changes in temperature before an actual stress event may enhance survival. Circadian control of transcriptional responses to low temperature has been observed (Fowler et al. 2005; Dong et al. 2011). In grapevine fruit *Vitis vinifera*, the transcriptional response to heat stress differs between night and day (Rienth et al. 2014, 2016). While many elements of the fundamental heat shock response pathway are well understood, the gating effect of the time of day and the influence of the circadian clock on this pathway remain understudied areas of research. The circadian clock gates specific temperature-related functions like thermomorphogenesis through a time of day dependent regulation of gene expression (Quint et al. 2016; Zhu et al. 2016). However, Zhu et al. (2016) did not observe gating of HSP70 expression and therefore suggest that the circadian clock itself is not involved in primary temperature perception.

The goal of this study was to determine the extent to which heat shock survival and the genome-wide transcriptional response to heat shock are gated by the time of day the stress is applied. Here we characterize the diel and circadian effects on the physiological and transcriptional response to heat stress. We present a comprehensive transcriptional analysis of *Arabidopsis* heat shock responses at different times of the day. We show that the time of day is a critical factor affecting both basal and acquired thermotolerance. We further refine the heat shock transcriptional response networks, identifying unique transcriptional responses at each time of day. These time of day differences can be used to understand how the regulatory networks driving heat stress response change throughout the day. By evaluating the response to heat stress at two times of day, we have identified where the existing heat stress signaling networks may be missing components, thus expanding our understanding of the signaling pathways that perceive and respond to heat stress.

## Results

### Survival in response to heat shock is influenced by the time of day when the heat shock is administered

**Fig. 1:**
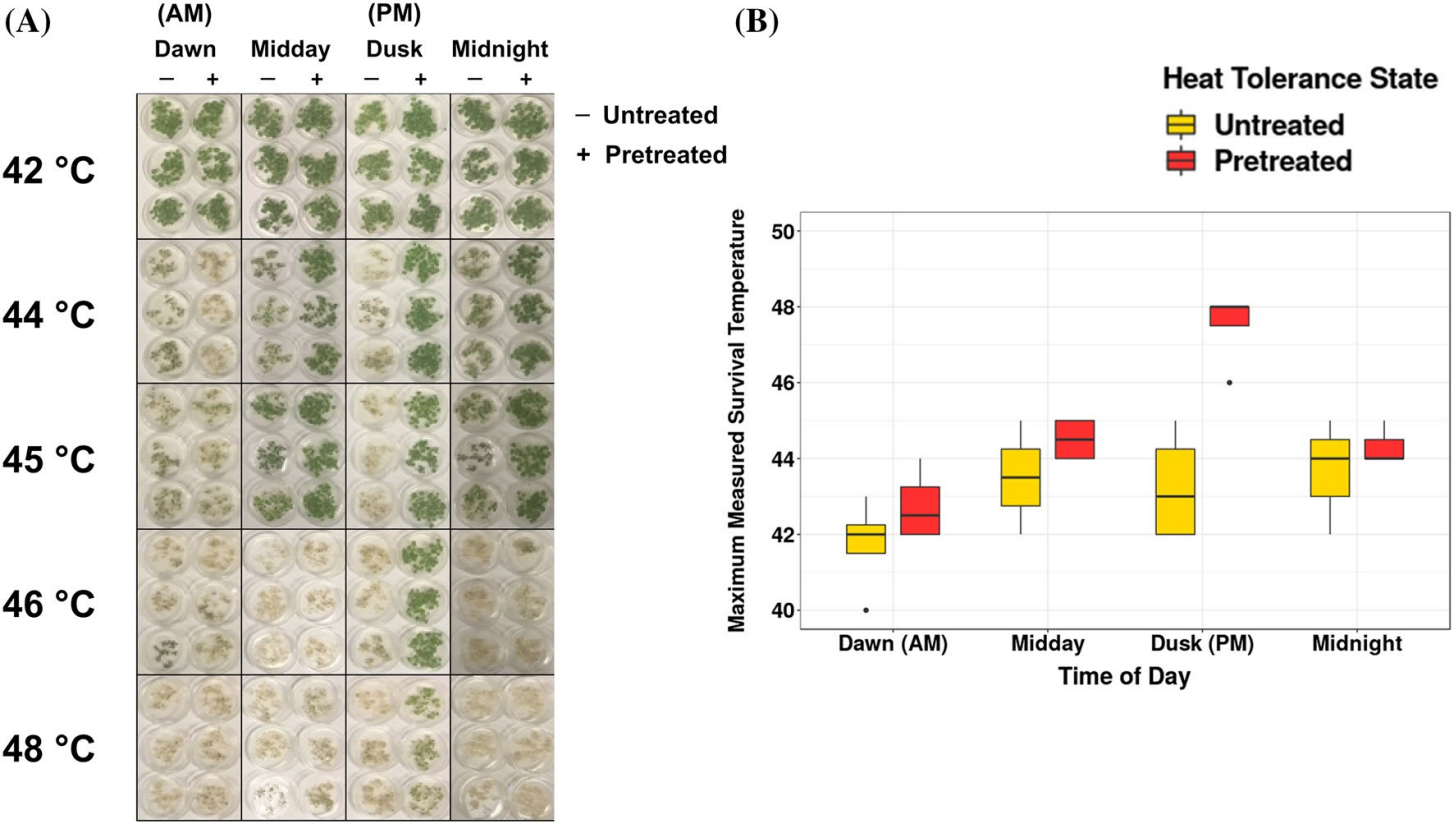
Time of day influences heat stress tolerance in both acclimated and non-acclimated *Arabidopsis* seedlings. **a** Single replicate experiment of *Arabidopsis* seedling heat tolerance after 1 h acclimation at 37 °C across all times of day (AM (Dawn)—ZT 1, Midday—ZT 7, PM (Dusk)—ZT 13, Midnight—ZT 19). Each black-outlined box contains six plates, with and without acclimation at each time point. A single picture was taken of all the plates. This picture was cropped around groups of plates and recombined to compress the image. **b** Box and whisker plots of survival data from four replicate *Arabidopsis* seedling heat tolerance experiments (n = 4 for AM, Midday, PM: n = 3 for Midnight). Range of heat shock temperatures tested across the experiments is 40–50 °C. Maximum measured 50% survival rate is quantified by recording the highest temperature at which 50% of seedlings regenerate aerial tissue

To evaluate if the time of day affects the response to both basal and acquired heat tolerance, we tested tolerance to heat shock in *Arabidopsis* seedlings with and without acclimation by exposing plants to a range of heat stresses at four different times of day: 1 h after lights on (AM), midday, 1 h after lights off (PM), and midnight. Acclimated plants were placed at 37 °C for 1 h and allowed to recover for 1 h before the heat shock was applied. We quantified plant survival by observing the temperature at which at least 50% of the plants survived after 1 h heat shock and 3 days of recovery. For both non-acclimated and acclimated plants, heat shock treatment at dusk resulted in survival at the highest temperatures (Fig. 1). A 1 h heat shock at dusk after 1 h of acclimation consistently resulted in plants that survived up to 46–48 °C, the highest temperature we tested. In contrast, in all experiments, the maximum temperature where survival occurred at dawn, midday, or midnight, was 46 °C, with most experiments resulting in lower acclimation temperatures. Across all experiments, we observed that the non-acclimated survival temperature was the lowest in the AM time point. Even without acclimation, the survival temperatures in the PM were in the same range as the acclimated survival temperatures in the AM (Fig. 1b). Our results show that plant thermotolerance varies across different times of day, with the greatest potential for survival at our PM time point.

To evaluate if the circadian clock controls this time of day difference in acclimation, we compared the heat stress response of plants at four times of day after transfer to continuous light. Plants were grown in 12 h:12 h light:dark cycles and then were shifted to constant light conditions. On the second day in continuous light, the plants were treated with a temperature stress 1 h after subjective dawn (AM), 6 h after subjective dawn (midday), 1 h after subjective dusk (PM), or 6 h after subjective dusk (midnight). We observed that in continuous light, plants treated at the PM time point survived higher temperatures both with and without acclimation compared to plants treated in the AM (Online Resource 1 Fig. S1). This conserved response in the absence of a day–night light cycle suggests that the circadian clock controls some portions of the observed difference in response to heat shock at different times of the day.

### The time of day when heat shock is applied affects the early transcriptional responses

In diel conditions, up to 80% of the *Arabidopsis* transcriptome shows a rhythmic pattern of expression (Smith et al. 2004; Bläsing et al. 2005; Michael et al. 2008). We reasoned that this underlying difference in the basal transcriptional levels could affect the transcriptional response to heat stress and lead to the variation in thermotolerance we observe (Fig. 1). We examined the expression in unstressed conditions of known heat shock response regulators (HRRs) and heat responsive genes (HRGs) in published diel and circadian datasets. The peak of expression of HRRs was widely distributed throughout the day and night in both diel and circadian conditions (Online Resource 1 Fig. S2). We also found HRRs are enriched for cycling genes (Online Resource 1 Fig. S3 a), while HRGs are enriched for peaks in expression at specific times of day (Online Resource 1 Fig. S3 b). This variation in unstressed conditions may affect the downstream signaling cascade and explain the temporal sensitivity to heat shock.

**Fig. 2:**
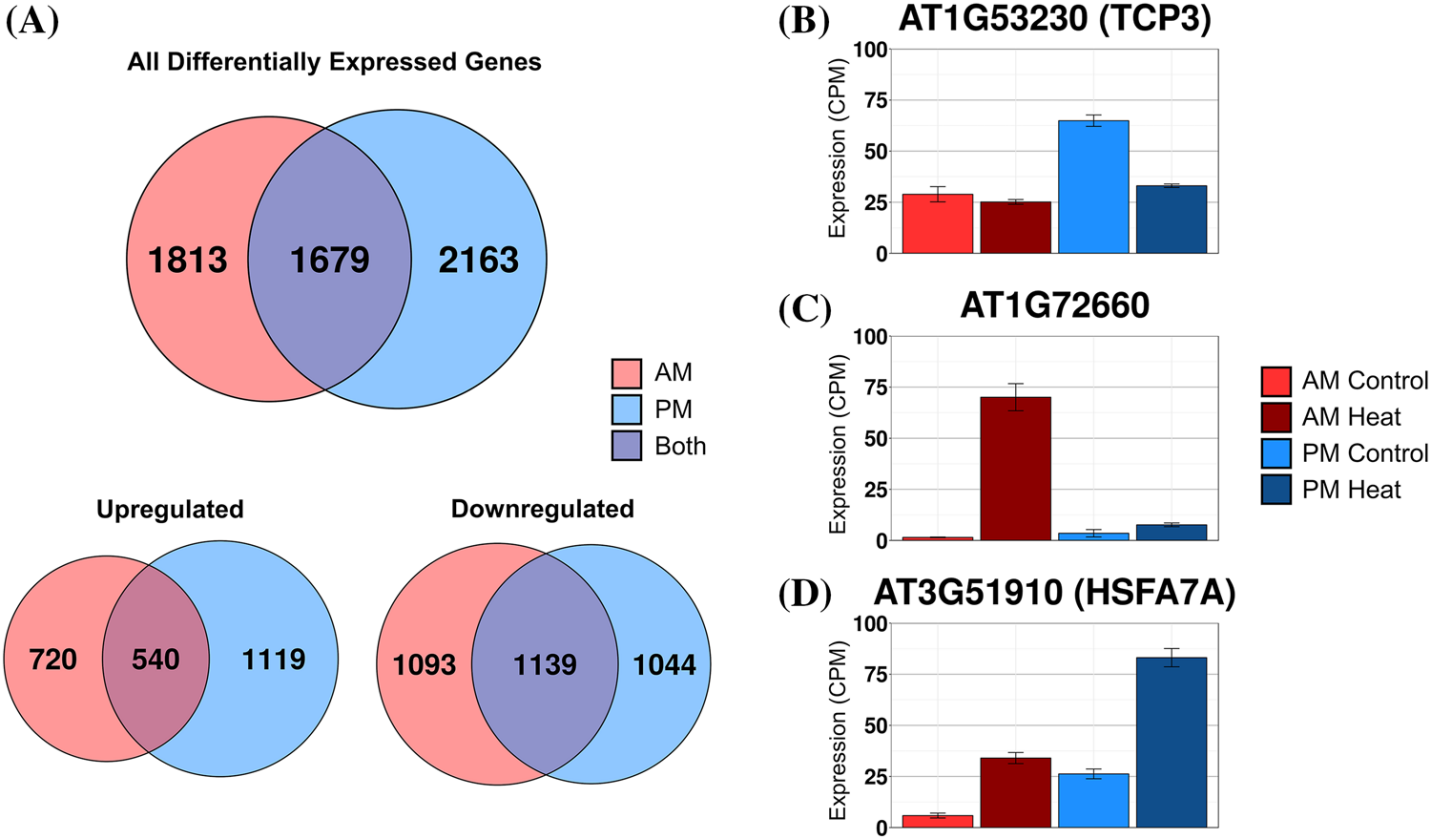
Standard categorization of *Arabidopsis* transcriptional response to heat shock. **a** Venn diagrams with the number of differentially expressed (DE) transcripts at each time of day (Log Fold Change > 0.5, adjusted *p* value < 0.05). **b** Expression levels of example transcript, AT1G53230 AM control (Light Red), AM heat shock, (Dark Red), PM control (Light Blue), PM heat shock (Dark Blue) demonstrating the effect of differences in control conditions on DE calls between the two time points. **c** Example transcript, AT1G72660 demonstrates circadian gating, where the magnitude of the induction in response to heat is substantially higher in the AM vs. PM time point. **d** Example transcript, AT3G51910 demonstrates both gating and basal differences. Error bars represent SE

To evaluate the global effects of time of day on the transcriptional response to heat shock, we examined the transcriptional response to heat stress at two times of day by RNA-Seq. We compared the transcriptional response to heat shock between plants treated 1 h after lights on (AM) and plants treated 1 h after lights off (PM) since we observed the most substantial variation in survival at these two time points (Fig. 1b). The lowest pretreatment temperature that leads to acquired thermotolerance in 1 h reported in the literature for *Arabidopsis* seedlings was 30 °C (Larkindale and Knight 2002). We confirmed that this moderate temperature stress induced acquired thermotolerance in our conditions. We exposed *Arabidopsis* seedlings to a 30 °C heat shock in either the AM or the PM time points for 1 h and quantified the transcript levels by RNA-Seq. Data for individual transcripts can be visualized at http://go.ncsu.edu/clockworkviridi. Heat-responsive transcripts were identified using DeSeq 2 (See “Materials and methods”, Analysis 1). Using a standard categorization approach that compares response in the AM to the PM we identified 5655 transcripts that were responsive to heat stress in the AM, PM, or both time points (Fig. 2a, Online Resource 2). Among these, 2379 were upregulated, and 3276 were downregulated. Enrichment analysis of each group indicated that heat shock and stress response GO categories are enriched in the uniquely AM responsive genes and genes responding at both times of day, but not in genes responding only at night (Online Resource 3). In transcripts responding specifically to the PM treatment, metabolic and cellular regulatory functions are enriched including response to stimulus, response to light intensity, DNA metabolic processes, and cell cycle processes (Online Resource 3).

### Temporal sensitivity of the early transcriptional responses to heat shock

We examined the transcriptional patterns across both the AM and PM time points to understand how the time of day could lead to different transcriptional responses. We considered transcripts to be gated if they showed a statistically significant different response to the same heat stress at the two times of day (e.g., Fig. 2b, c). Gating differences in heat shock responses could be observed if the transcript starts at different levels in unstressed conditions, shows a time of day sensitivity in response to heat stress, or a combination of both effects (e.g., Fig. 2d). Classifying genes based on which of these mechanisms lead to their difference in expression between the two times of day will facilitate understanding the regulatory differences that drive the variation in the transcriptional response to heat stress.

**Fig. 3:**
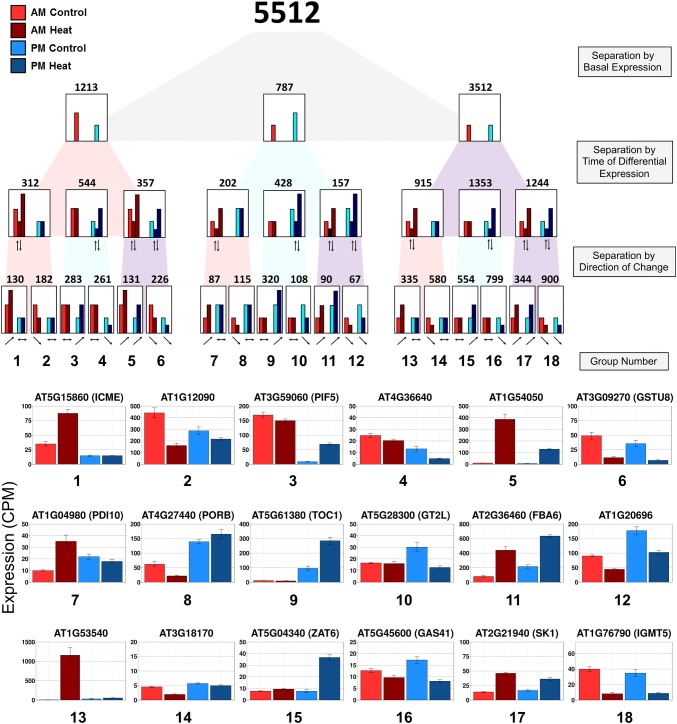
Refined categorization of differentially expressed genes responding to heat shock. Model diagram of how the refinement separates heat shock responsive genes at each branch point based on their expression level in AM control (Light Red), AM heat shock, (Dark Red), PM control (Light Blue), PM heat shock (Dark Blue) into eighteen subcategories. Each bar represents example expression of each time point for genes filtered at each step. Row one separates the 5512 heat responsive transcripts by their basal expression, grouping genes based on if their expression in unstressed conditions is higher in the AM (left, 1213); PM (middle, 787); or not differentially expressed between these two times of day (right, 3512). The relative height of the light-colored control bars represents the difference in unstressed levels between AM (red) and PM (blue). The second row separates the transcripts further based on if they are differentially expressed in the AM, PM, or at both times of day. The darker colored bars indicate the response to heat stress in the AM (dark red) or in the PM (dark blue). In this row, the transcripts have not been separated based on the direction of the response and where the dark colored bars are double indicates that the transcripts in that group are either up or down regulated at that time point. The bottom row separates the transcripts further by the direction of the response, splitting the genes into upregulated and downregulated categories, with arrows indicating the direction of differential expression. The numbers above each category are the total number of genes within the category at that level of the categorization. Categories with less than ten genes were omitted. Normalized expression levels for example genes for each category, identified by the corresponding category number are shown below. Error bars represent SE

Gated genes may result from different regulatory activity, such as the presence or absence of specific activators or repressors, at the two times of day. To clearly identify and categorize AM and PM gated transcriptional responses we developed a refined categorization approach that classifies the heat response of each transcript based on three criteria: (1) The differences in expression in unstressed conditions between AM and PM, (2) If the response to heat shock is unique to one time of the day, and (3) If the heat response is up- or downregulated (Fig. 3). Using DeSeq 2, we analyzed the samples from all time points combined (See “Materials and methods”, Analysis 2 for linear model parameters). We identified 5512 transcripts that showed a response to heat in either time point. In unstressed conditions, 1213 (22% of all heat responsive transcripts, categories: 1–6) had a higher AM basal level and 787 (14%, categories: 7–12) had a higher PM basal level. The remaining 64% (3512 genes, categories: 13–18) had similar expression levels in both AM and PM under unstressed conditions. We further divided each of these three categories based on whether they responded to heat only in the morning, only in the evening, or equally at both times of the day. Finally, transcripts were separated based on if the transcript was induced or repressed in response to heat. This classification results in 18 categories with 10 or more transcripts per category (Online Resource 4).

Time of day independent transcripts with no temporal differences in the unstressed levels or the heat shock response between the AM and PM treatments account for 23% of the heat-responsive transcripts. These 1244 transcripts (categories: 17–18) include previously identified heat shock responsive factors such as HSFA2, MBF1c, and HSP26.5 (Sakuma et al. 2006; Nishizawa et al. 2006; Suzuki et al. 2008) (Online Resource 1 Fig. S4). We also considered as time of day independent the 514 genes (9%, categories: 5–6, 11–12) with different basal levels, but a similar induction or repression in response to heat at both times of day (Fig. 3). This group includes heat-responsive factors such as HSP90.1, HSFB2A, and HSFA7A (Nishizawa-Yokoi et al. 2011; Cha et al. 2013; Lin et al. 2018).

Not all classic heat shock response factors are time-independent. For example, we observed that APX2 (AT3G09640) and many of the HSP20-like transcripts (e.g., AT1G53540), are maintained at low basal levels at both times of day but are strongly upregulated in response to heat only in the AM (Fig. 3, Online Resource 1 Fig. S4). We observe that 3754 heat-responsive transcripts (68%, categories: 1–4, 7–10, 13–16) show a differential response depending on the time of day the heat shock is applied, meaning these responses are gated. Many of these heat-responsive transcripts have similar AM and PM expression levels in unstressed conditions yet still show a temporal sensitivity to the heat stress. These 2268 transcripts (41%, categories: 13–16), respond either only in the AM (915, categories: 13–14) or only in the PM (1353, categories: 15–16) (e.g., AT1G53540—*HSP20*-*like* and *ZAT6*) (Fig. 3). We also observed combined effects of both differences in basal levels and the response to heat stress for 1486 (27%, categories: 1–4, 7–10) of the transcripts. Of these transcripts that start at different basal levels, 514 (9%, categories: 1–2, 7–8) show a response to heat stress only in the morning. While 972 (18%, categories: 3–4, 9–10) start at different basal levels and show a response to heat stress only in the evening, consistent with our observation that more uniquely heat-responsive transcripts are detected in the PM time point (Fig. 2a).

A subset of gated transcripts highlights the fact that examining the fold-change in response to heat stress may not provide a full characterization of the transcriptional response. For these transcripts, the level of transcript accumulation in unstressed conditions must also be considered. These transcripts are gated, showing a response to heat stress at only one time of day. However, the induction or repression after heat stress may not be significant when compared to the unstressed condition at the other time of day. For example, TCP3 transcripts (Fig. 2b) are significantly reduced in response to heat stress in the PM timepoint where the basal level is higher. In the AM time point, where the basal expression is lower, the TCP3 response to heat stress is not significant. Similarly, PIF5 is not induced under heat stress in the AM. However, in the PM, where the unstressed expression is significantly lower than the AM unstressed levels, the response to heat in the PM time point is significant (Fig. 3). For these transcripts, comparing the changes in absolute level will result in a different interpretation than comparing the fold change (Online Resource 1 Fig. S5). In total, there are four categories with 660 transcripts (12%, categories: 2, 3, 7, 10) that have this pattern of expression where the time of day differences in response are primarily due to the differences in expression under unstressed conditions.

### Functional enrichment of heat responsive transcripts varies depending on the time of heat treatment

**Fig. 4:**
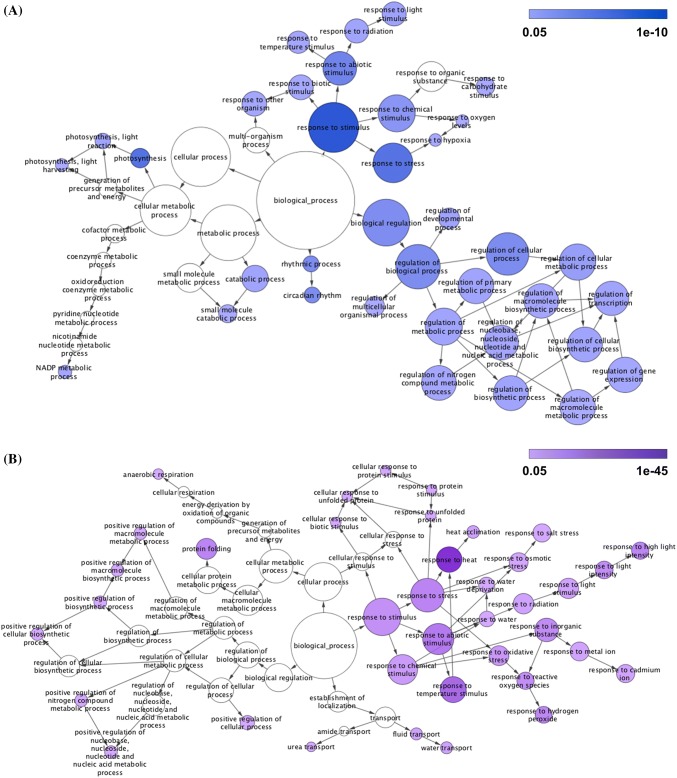
Time specific GO functional response networks. **a** The GO enrichment network for uniquely PM responsive transcripts. Functional categories upregulated only in the PM are colored blue and shaded based on the level of significance. **b** The GO enrichment network for transcripts that are heat shock responsive at both times of day. Functional categories upregulated at both AM and PM are colored purple and shaded based on the level of significance. We did not detect functional enrichment for any GO categories for transcripts upregulated only in the AM

Given that the transcriptional heat response showed significant variation depending on the time of day, we evaluated if there is a fundamental difference in the biological pathways involved in the response in the AM versus the PM. We analyzed functional enrichment from the refined categorization using gene ontology (GO) annotations (Ashburner et al. 2000; Carbon et al. 2017). Using BiNGO, we generated GO networks for genes that were upregulated only in the AM or PM or at both times of day (Maere et al. 2005). We did not detect any functional enrichment for genes uniquely upregulated in the AM. We then generated two networks of enriched functions: one for genes upregulated uniquely in the PM (Fig. 4a) and one for genes upregulated at both times of day (Fig. 4b). The majority of the categories related to heat shock were enriched in the transcripts induced at both times of the day, including “response to heat,” “protein folding”, “heat acclimation”, “water transport”, and “response to reactive oxygen species.” Since these GO terms are enriched in the categories that respond at both times of day, they may not contribute to the difference in survival and acclimation observed between the AM and PM samples. However, the transcripts responsive only in the PM are enriched for many uniquely night-responsive functional categories including photosynthetic and metabolic regulation. These pathways are transcriptionally activated in response to heat stress only in the PM either because these pathways are already expressed in unstressed conditions in the AM or because the response is gated.

We also investigated the GO functions enriched in downregulated transcripts (Online Resource 1 Fig. S6). In genes downregulated at both times of day (Fig. S6A), we observe general stress response and metabolic categories like “secondary metabolic process” and “cellular amino acid and derivative metabolic process.” Uniquely PM repressed transcripts (Fig. S6 b) are enriched for “DNA metabolic process” and “cell cycle.” Terms enriched in genes repressed only in the AM (Fig. S6 c) include “protein modification process” and “carbohydrate metabolic process”. This indicates that although there is a time-independent repression of some metabolic processes, both AM and PM time points contain repression of unique functions which can contribute to the difference in response to heat shock at each time of day we observe. This high-level overview of differences between dawn and dusk provides functional categories and associated genes that may connect to the differences seen in physiology and plant survival after heat stress. We collected in-depth GO data across all of our 18 categories using the PANTHER database (Thomas et al. 2003; Mi et al. 2017). We analyzed all detected functional enrichment groups and identified a total of 35 pathways (available at http://go.ncsu.edu/clockworkviridi) (Online Resource 5).

### Transcripts responsive at different times of day are enriched for different *cis*-regulatory elements

To understand the transcriptional regulatory cascade which drives the gated response to heat shock we observe, we analyzed promoter *cis*-regulatory motifs to identify time of day specific features. We searched for enriched *cis*-regulatory elements using Homer2 (Heinz et al. 2010) (Online Resource 6). A total of ten enriched elements were detected (*p* value < 1e–10) for genes responding at both times of day (e.g., AtMYB84-binding, AtHB5-binding). Additionally, we detected 4 enriched elements specific to AM responsive genes (e.g., bHLH-binding, Online Resource 6), and 5 elements specific to PM responsive genes (e.g., bZIP binding, E-box, Online Resource 6). We hypothesized that separating heat-responsive genes by the time of day they respond based on both basal levels and response to heat shock might reveal novel regulatory features. For example, transcripts such as TCP3 (Fig. 2b), which respond to heat shock only at night, may have been missed in previous analysis of *cis*-regulatory elements involved in heat responses. We identified enriched *cis*-regulatory motifs for gene categories identified by either the standard or refined categorization methods. We compared the enriched motifs identified in the standard categorization to our refined method which incorporates the difference in basal expression levels. Some motifs are detected in both approaches (e.g., HSE binding site motif AGAANNTTCT, standard: *p* value < 1e−90 refined: *p* value < 1e−61). In PM-responsive genes, we identified a bZIP transcription factor binding element using the standard approach (CCACGTCAKC, (*p* value < 1e−19). This motif was also enriched in category 9 using our refined classification (*p* value < 1e−12). The refined classification provides additional information on the expression patterns of the genes containing this motif. The expression pattern of the genes in this cluster are upregulated in the PM, but also have higher PM basal levels which means this element could also be involved in gene regulation in unstressed conditions. The refined categorization also identified enriched motifs that are not detected in the standard approach. We detect an unknown element (CGATTCACAC, *p* value < 1e−11) in category 15. This element is not enriched in the standard categorization in any group of differentially expressed genes, including those upregulated in the PM. In total, we detected 12 unique elements in the refined categorization, and 10 unique elements in the standard categorization. While each approach identifies different elements, the finer tuning of the refined categorization, where there are fewer genes in each category and specific separation of categories based on expression in unstressed conditions and in response to heat shock, provides motifs that account for a specific expression pattern.

### Heat shock response in *Arabidopsis* is gated by the circadian clock

**Fig. 5:**
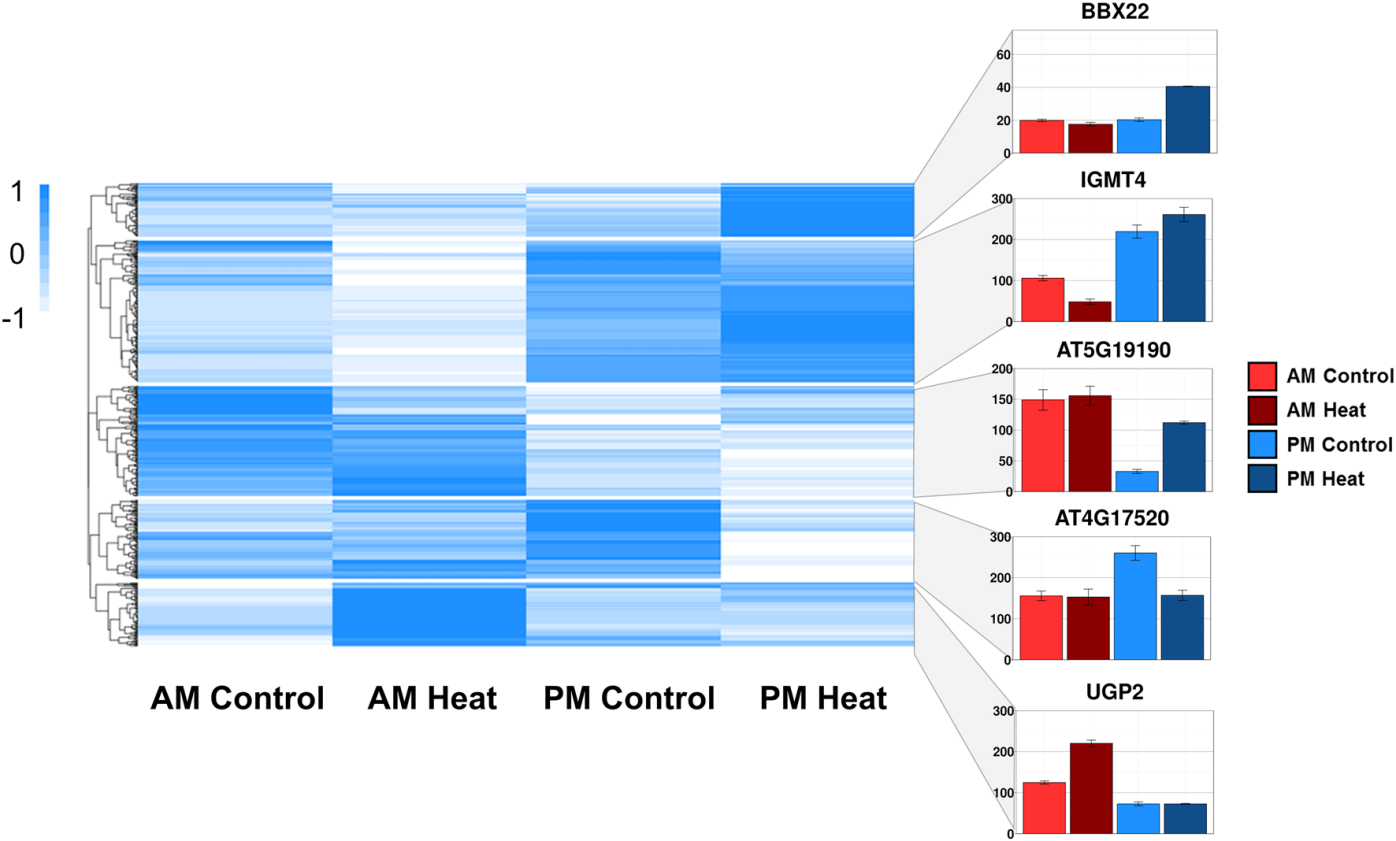
Heatmap of temporally heat responsive transcripts. We performed hierarchal clustering on the 572 temporally heat responsive transcripts. Each gene is scaled independently by row, the color represents the level of normalized gene expression, ranging from blue (highest expression level) to white (lowest expression level). Five primary groups, representing time of day dependent heat shock response patterns are highlighted. Expression levels in AM control (Light Red), AM heat shock, (Dark Red), PM control (Light Blue), PM heat shock (Dark Blue) are shown for a representative gene for each group, y-axis of the inset example genes is Expression levels (CPM). Error bars indicate SE

To identify the heat-responsive transcripts that are the most affected by the time of day, we performed differential expression analysis with a linear model that considers both the condition (untreated vs. treated with heat) and the time of day (day vs. night) (See “Materials and methods”, Analysis 3 for detailed linear model). We classify the resulting 572 differentially expressed genes as our temporally heat responsive transcripts. Using hierarchal clustering, we grouped these genes to identify representative response groups (Fig. 5, Online Resource 7).

Clustering reveals five major patterns of heat-shock responses: Two groups that are upregulated in the PM (groups 1 and 3), downregulation in the AM (group 2), downregulation in the PM (group 4), and finally AM upregulation (group 5). Group 1 and group 3, the two PM upregulated groups, are separated by basal expression levels. Group 3 contains transcripts with higher AM basal levels than group 1, although both are strongly upregulated in response to heat in the PM. In this set of 572 differentially expressed genes, we identified 78 transcription factors. Among these, the pseudo-ARR transcription factors are enriched (adjusted *p* value < 0.05), suggesting they play a role in the time of day variation in heat responses.

**Fig. 6:**
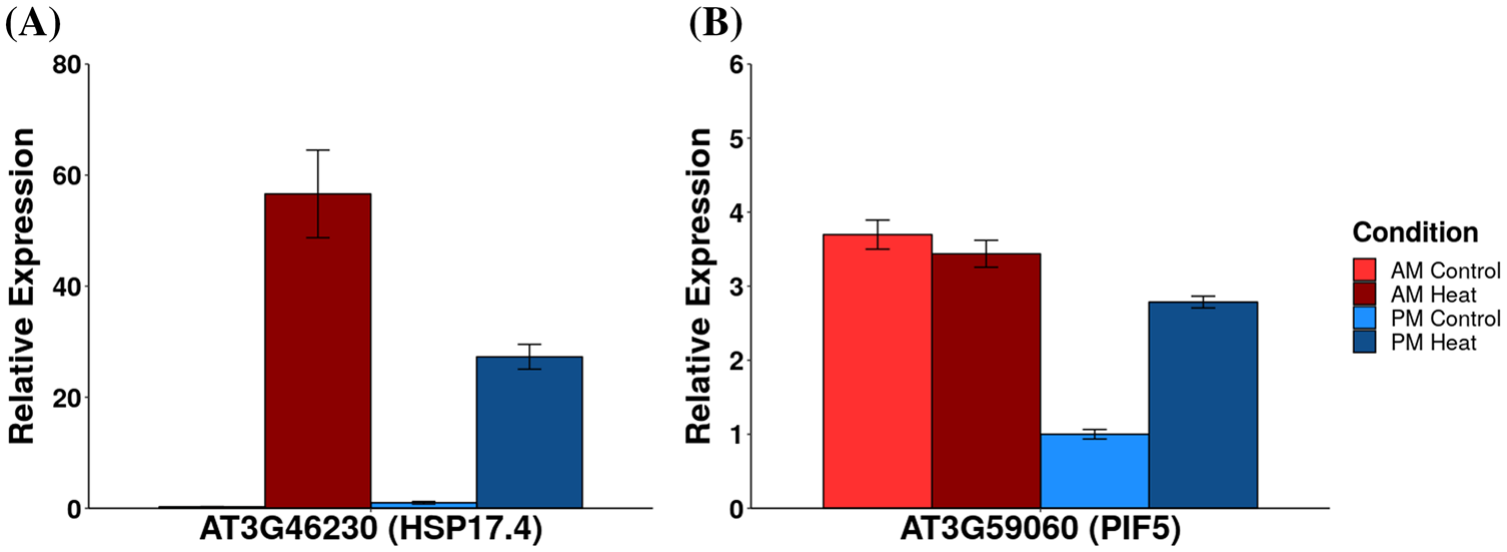
Expression of gated genes in constant light. qRT-PCR analysis of plants grown in 12:12 light:dark cycles and then transferred to constant light. On the second day in constant light, plants were heat shocked 1 h after subjective dawn (AM) or 1 h after subjective dusk (PM). **a** Expression levels of AT3G46230 (*HSP17.4*) in AM control (Light Red), AM heat shock, (Dark Red), PM control (Light Blue), PM heat shock (Dark Blue) normalized to the PM control expression level. **b** Expression of AT3G59060 (*PIF5*) in constant light conditions normalized to levels in the PM control samples. Error bars represent ± standard error, n = 4

To evaluate the role of the circadian clock on the observed transcriptional responses and the effects of gating we tested the heat shock response in the absence of diel cues in constant light. We grew *Arabidopsis* seedlings in 12 h light:12 h dark at 23 °C and transitioned the chamber into constant light and temperature conditions after 10 days. At 12 days, we heat shocked the plants at 30 °C for 1 h after subjective dawn and 1 h after subjective dusk and compared control and heat-shock gene expression levels by RNA-Seq and qRT-PCR. For two transcripts which were classified as either gated (*HSP17.4*, category 17) or induced only in the PM (*PIF5*, category 3) in response to heat shock in diel conditions, we examined the response in constant light by qRT-PCR with four replicates. *HSP17.4* is induced in both the AM and PM time points in constant light (Fig. 6a). However, the level of induction is still greater in AM (*t* test, *p* value < 0.05). The evening specific transcriptional response of *PIF5* to heat shock persisted in circadian conditions. As we observed in light:dark cycles, under continuous light, expression of *PIF5* is high in the AM control plants, and there is no significant response to heat shock in the AM. In the PM, *PIF5* expression is low in control conditions, and a significant increase in response to heat shock occurs, returning *PIF5* to the AM control levels (Fig. 6b). Like *PIF5*, the time of day difference in response for the 660 transcripts in categories: 2, 3, 7, 10 is primarily due to the differences in expression between AM and PM time points in unstressed conditions. In previous analysis of transcriptional responses in constant light (Harmer et al. 2000; Edwards et al. 2006), the unstressed levels of these 660 genes retain their variation in expression throughout the day and are enriched for cycling genes (*p* value < 1e−12 for both circadian datasets). To determine if the trend in time of day variation of transcriptional response persisted, we analyzed one replicate from each time point by RNA-Seq. Of the 572 time-dependent transcripts (Fig. 5), 191 (33%) showed a similar gating response in constant conditions (Pearson’s correlation coefficient between the circadian and diel data > 0.7). We manually compared transcripts with a correlation coefficient between 0.5 and 0.7 and observed that another 18 transcripts showed a similar trend in transcriptional response to heat stress between the diel and circadian experiments. Data for individual transcript expression in constant light can be visualized at http:/go.ncsu.edu/clockworkviridi. The conserved gating effects under constant light (Fig. 6) indicate that the circadian clock is partially involved in regulating the transcriptional processes that lead to the time of day differences in the transcriptional response to heat shock. These results are consistent with the persistent differences in survival between dawn and dusk we observe in constant light (Online Resource 1 Fig. S1).

## Discussion

### Temporal variation in physiological and transcriptional responses to heat stress

Examining the transcriptional response of *Arabidopsis* to heat shock at two times of day offers a unique perspective on the complex interaction between time and temperature. We observed that both the transcriptional and physiological responses vary depending on the time of day when the stress is applied. *Arabidopsis* plants showed the greatest tolerance to heat shock when the stress was given 1 h after dusk. Plants treated 1 h after dawn had the greatest sensitivity to heat shock. There was little difference in either the basal tolerance or acclimated tolerance in plants treated at midday or midnight suggesting that it is not simply the presence or absence of light that contributes to this variation in response between dawn and dusk. Solar noon, the period when the sun’s radiation is the strongest, occurs around midday. However, in many locations the earth receives more heat than it can radiate back into space. This period of delay between solar noon and the maximal temperature known as the thermal response. Although a number of environmental factors impact the thermal response, in summer it can extend for several hours. The observations here of enhanced tolerance to heat stress at dusk compared to dawn, midday, or midnight are consistent with dusk being the hottest period of these four times during the summer months in many locations. Moreover, in plants grown in constant light, the variation in response observed in diel conditions persisted. These results suggest that this daily variation in sensitivity is in part controlled by the circadian clock serving to anticipate the hottest times of the day. Previous work has shown that light-induced chloroplast signaling is involved in the time of day dependent thermosensitivity that we observe (Dickinson et al. 2018).

### Time of day sensitivity in the transcriptional response to heat shock

Similar to the variation in survival between dawn and dusk, the transcriptional response to 1 h heat stress shows a different profile depending on the time of day the stress is applied. This indicates that the transcriptional response to a stress applied at a single time of day is only a snapshot of the range of possible responses. Evaluating the response at two time points reveals some challenges with transcriptional analysis that may not be obvious when examining the response from a treatment at a single time of day. For example, the most common method of identifying genes of interest in response to a stress is to identify those with a large induction compared to the control. However, a gene can be significantly induced by heat shock at one time of day compared to the control and yet the absolute level could be well below the unstressed level at the other time of day. For example, AT3G59060 (PIF5), is induced 7.9 fold in response to heat stress in the PM time point, but that heat-induced level is still significantly below (2.5 fold) the unstressed level at the AM time point (e.g., Online Resource 1 Fig. S5). We have only sampled two times points, yet 660 genes show this type of expression pattern (e.g., Fig. 3: Categories 2,3,7, and 10). Variation in unstressed levels at other times of day could be greater than the heat shock response we see at the two times of day we stressed the plants. The digital nature of RNA-Seq allows us to consider the absolute level of gene expression with a confidence not possible through hybridization analysis methods. This raises an important question: is the level of induction or the absolute level of expression the more relevant biological feature for gene function? This most likely depends on the question of interest. In our example, we speculate that the transcriptional induction of PIF5 at night may not be relevant to the enhanced survival. This is because we observe that its absolute level is higher in the morning in both unstressed and heat shock conditions, when the plant is more sensitive to heat, compared to the PM time point after heat shock. However, for identification of transcriptional regulators and conserved *cis*-regulatory elements, it is important to focus on the response to heat shock. Increases in the level of these transcripts, regardless of the absolute level, may indicate that the transcript is downstream of a pathway that senses heat and therefore, can be used to identify upstream components. It is important to note that our RNA-Seq approach is only measuring the steady state levels of RNA, therefore what we interpret as an induction could also be a consequence of changes in turnover (Lidder et al. 2005; Ding et al. 2014; Su et al. 2018).

To examine the regulatory differences between the two times of day, our approach focused on the change in levels compared to the unstressed controls, ignoring differences in the absolute level. We used a time-based supervised categorization to classify differentially heat-responsive transcripts. By first considering the underlying changes in the control samples across time, this approach identifies genes that show temporal variation in their response to heat shock, distinguishing genes with gated responses from those that are independent of the time of day. Motif enrichment analysis improved when analyzing categories from the refined categorization. Expected motifs, such as the heat shock element, could be detected in both methods. However, our ability to detect novel motifs when grouping into finer categories based on both factors indicated that the basal expression level and the response of the gene provide new insights when considered in combination.

### Time of day specific transcriptional signature in response to heat stress

A more complete understanding of the heat shock response requires that we consider temporal effects. It has previously been suggested that different abiotic stresses such as heat and salinity produce unique molecular signatures (Martinez et al. 2016). For example, each stress produces a unique ROS signature and this classification can help identify specific tolerance mechanisms. We propose that this signature of response can be extended to include a temporal component that has been mostly ignored. We observe that time of day the heat shock is applied can alter the heat-response signatures. Like stress-specific signatures, this variation can be used to understand the network connections and facilitate comparing experimental results between laboratories. In our analysis of the response to heat shock at dawn and dusk, we observe differences in the downstream transcript levels. This observation suggests that the *Arabidopsis* molecular heat response network varies between these two times of day.

HSFA1 factors serve as master regulators of the heat shock response (Nishizawa-Yokoi et al. 2011). HSFA1a, HSFA1e, and HSFA1d are not differentially expressed in response to heat shock at either time point, in agreement with the mechanism that HSFA1s are activated by release of the repression activity of HSP70 and HSP90 (Yamada et al. 2007; Hahn et al. 2011; Ohama et al. 2015). We observe HSFA1b is induced in the PM, but not in the AM. Yoshida et al., (2011) identified candidate HSFA1 targets by examining the change in the transcriptional response to heat stress in the *hsfa1a/b/d/e* quadruple mutant (Yoshida et al. 2011). Sixteen of their top 100 targets were molecular chaperones. We observed induction of all 16 of these molecular chaperones and the response was higher in the AM, for all l6 transcripts, with four showing little or no induction in the PM (e.g., AT1G53540 and AT4G27670 – *HSP21*) perhaps suggesting that the transcriptional induction by HSFA1s is enhanced by other factors present in the AM (Fig. 3, Online Resource 1 Fig. S4). However, another target of HSFA1, DREB2A, is induced by heat shock in both the AM and PM, with a larger induction level in the PM (Yoshida et al. 2011) (Online Resource 1 Fig. S4). This difference in the temporal response of the targets of HSFA1 suggests additional components may function in combination with HSFA1 to provide specificity in the induction of the HSFA1 targets. There are other known genes in the heat shock response which are induced at both times of day, including HSP70, HSP90.1, and HSP90.2 chaperones. These three HSP factors are also induced to a higher level in the AM compared to the PM (Online Resource 1 Fig. S4). Known transcription factors HsfA7A, HsfA7B, and HsfA2 are also upregulated at both times of day, with HsfA7A induction in the PM significantly higher than the AM (Online Resource 1 Fig. S4). Other factors in this network show temporal gating to a specific time. DREB2C, and HsfA3 show upregulation specifically in the PM (Online Resource 1 Fig. S4). Some of these targets with time-specific responses are strong candidates for connecting transcriptional regulation to the variable physiological response to heat shock we observed.

MBF1c, another upstream regulator of transcriptional response to heat stress, is induced in response to heat stress in both AM and PM treatments (Online Resource 1 Fig. S4) (Suzuki et al. 2005, 2008). The MBF1c regulon has been previously defined (Suzuki et al. 2011). In our data, these MBF1c targets can be further divided. There are those that like MBF1c show a response at both times of day (e.g., AT2G27580 AN1-like, Online Resource 1 Fig. S4). However, some MBF1c regulon genes show a temporal difference in their response. The MBF1c target gene AT2G44130 (Kelch repeat F-box protein) is upregulated only in the PM, while AT5G05410 (DREB2A) responds at both times of day with a higher induction in the PM (Online Resource 1 Fig. S4). Some MBF1c targets show an inverted response, repressed in response to heat stress in the AM and induced in response to heat stress in the PM (e.g., AT3G13310, DNAJ Heat shock protein, Online Resource 1 Fig. S4). This temporal variation in the response of MBF1c regulon genes may indicate other factors are required to regulate these targets in collaboration with MBF1c.

### Implications for acquired thermotolerance and heat stress memory

Heat stress memory is the capacity for plants to acquire long-term thermotolerance after exposure to a heat stress event (Charng et al. 2007; Wu et al. 2013). Two components of heat stress memory, *HSFA2* (AT2G26150) and *ROF1* (AT3G25230) respond robustly in response to heat shock in both the AM and PM (Online Resource 1 Fig. S4) (Nishizawa et al. 2006; Charng et al. 2007; Meiri and Breiman 2009). The HSFA2 targets classified as heat-shock inducible memory genes *HSP21* (AT4G27670), *HSP22 (*AT4G10250*), HSP18.2* (AT5G59720), and *APX2* (AT3G09640), are all induced significantly in response to heat stress in the AM, with either no response or a very minimal response to heat stress in the PM (Online Resource 1 Fig. S4). The lack of induction of these genes in the PM, despite the high mRNA levels of *HSFA2* at both times indicates that *HSFA2* expression alone is not sufficient to induce the heat-response of these transcripts. This is consistent with previous analysis demonstrating that HSFA2 is not responsible for the early response of these genes to heat stress (Lamke et al. 2016). In contrast, the non-memory genes HSP70 (AT3G12580) and HSP101 (AT1G74310), are significantly induced at both times of day, although the absolute count levels in response to heat in the PM are substantially lower than the levels in the AM. Similarly, HSA32 (AT4G21320), a factor important for heat shock memory, is a heat shock protein unique to plants and some microorganisms (Charng 2006; Lamke et al. 2016). *HSA32* responds robustly at both times of day, but the AM heat shock induction is 36.2 fold, while induction in the PM is only 5.5 fold. (Online Resource 1 Fig. S4). These time-dependent variations in the response of heat stress memory genes suggest that time of day may affect heat stress memory. We observe that the time of day has a significant impact on the level of acquired thermotolerance. Given these temporal differences in some components of heat stress memory, it will be interesting to examine if the time of day that acclimation occurs has an effect on heat stress memory.

### Daily temperature sensing mechanisms and relationship to heat shock

Daily light and temperature signals are integrated and connected to growth in *Arabidopsis* through regulation of the Phytochrome Interacting Factor (PIF) family by Phytochrome B (PHYB) (Franklin and Quail 2010; Jae-Hoon et al. 2016; Legris et al. 2016; Qiu et al. 2019). *PHYB* regulates thermo-responsive growth through interaction with the PIF family, of which PIF1, 3, 4, 5, and 7 promote hypocotyl elongation (Leivar et al. 2008; Shin et al. 2009; Qiu et al. 2019). We observed significant differential expression for *PIF1*, *4* and *5*. *PIF1* is upregulated in response to heat shock at both times of day, while *PIF4* and *5* are upregulated specifically in the PM. The evening complex (EC), composed of ELF3, ELF4, and LUX, binds the promoters of *PIF4* and *PIF5* and represses the transcription of these targets (Nusinow et al. 2011). EC target binding is decreased during growth in higher temperatures (Ezer et al. 2017). The increased transcriptional activation of *PIF4* and *PIF5* at night during heat stress is consistent with the reduction of EC repression activity under higher temperatures (Ezer et al. 2017). To analyze downstream responses regulated by the PIFs under heat shock, we examined the expression of PIF4 and PIF5 target genes (*YUC8, IAA19, IAA29, TAA1, LNG1, LNG2*) (Koini et al. 2009; Franklin et al. 2011; Hao et al. 2012; Hwang et al. 2017; Qiu et al. 2019). *YUC8,* and *IAA19* are downregulated in the PM, *TAA1* is downregulated at both times of day, and *IAA29, LNG1,* and *LNG2* are not differentially expressed. Thus, these PIF targets are not being activated even though PIF4 and PIF5 expression is increasing under heat stress. TOC1 interacts with PIF4 and suppresses the activation of PIF4 target genes (Zhu et al. 2016). In our experiment, we observed significant upregulation of TOC1 in the PM heat shock sample which may explain why PIF4 targets such as *YUC8* are downregulated even though PIF4 is upregulated. It is likely that even though PIF4 and PIF5 are being activated by heat stress, other interacting factors in these regulatory pathways such as TOC1 are also being expressed in order to regulate PIF function, demonstrating the complexity of responses in this network.

### Commonly identified heat responsive transcripts are biased towards time-independent responses

To examine if the time of day contributed to variation in identification of heat-responsive genes between laboratories, we compared our differential expression results to previous high-throughput studies analyzing *Arabidopsis* response to heat stress. We identified two microarray studies which tested a similar exposure to heat stress compared to our experiment and filtered their differential expression results following the cutoffs applied to our data. Nguyen et al. exposed two-week old wild-type *Arabidopsis* to a 37 °C heat stress for 1 h and detected 12,891 differentially expressed genes in response to the heat stress (Nguyen et al. 2014). Shedge et al. exposed eight-week old wild-type *Arabidopsis* to a 37 °C heat stress for 2 h and detected 5479 differentially expressed genes (Shedge et al. 2010). The total number of differentially expressed genes overlapping between the two datasets is 3017, showing that although testing similar stresses, variability still exists in the transcriptional response. We hypothesized that this variability is in part because the groups may have performed their experiments at different times of the day. The times of day they performed their experiment were not available. We expect that the genes which we identified as time of day independent genes will be more likely to be identified by both groups. To test this possibility, we calculated the enrichment of our time-independent genes in the genes detected by both Nguyen et al. and Shedge et al. which overlap with our data (1205 total). We found that the genes detected by both groups are enriched for time-independent heat-response genes (*p* value < 1.1e−15). This suggests that the heat shock response genes in common between experiments performed by multiple groups may be biased towards time-independent responses. To evaluate if the genes identified by only one of the two groups were biased to a specific time of day, we examined the overlap between the uniquely detected genes by each group and our heat responsive genes with a high confidence interaction with time (572 genes, Fig. 5). We found that the heat responsive transcripts detected uniquely by Shedge et al. were enriched for AM specific response genes (*p* value < 0.02), indicating that the data may have been collected at a similar time of day to our AM time point. We found significant under-enrichment for both AM and PM specific response genes in the unique genes detected by Nguyen et al. (*p* value for AM genes < 3.0e−8, *p* value for PM genes < 0.01). A summary of these comparisons can be found in the supplemental data (Online Resource 8). Since the genes we identified as responsive based on the two time points we examined were not enriched in the Nguyen et al. study, this may indicate that this experiment was performed at a time far from the dawn or dusk time points we selected.

We hypothesized that temporal variability could drive the lack of consistency observed between in the transcriptional responses reported from different laboratories. We believe that a major reason that previous studies analyzing heat shock show variability is not only due to differences in treatment and environmental conditions, but also due to the time of day they perform the experiment. Our consideration of temporal effects illustrates the power of detecting time-dependent responses, since we are able to detect heat shock responses which have previously not been observed in heat shock studies in *Arabidopsis*. In support of this observation, no enriched GO terms were detected in the heat-responsive categories with higher expression in the PM in unstressed conditions (Fig. 3, categories: 7–12). In contrast, enriched GO terms were identified for all heat-responsive categories derived from transcripts with expression in unstressed conditions that was either higher in the AM or equally expressed in the AM and PM (Fig. 3, categories: 1–6 and 13–18, Online Resource 5). Categories of genes which respond at night contain the strongest candidates for novel heat shock response genes since few experiments are performed in nighttime hours.

### Temporal effects have a significant impact on heat shock responses

Circadian gating of stress responses has been previously observed in *Arabidopsis* for abiotic stresses including response to low temperature (Fowler et al. 2005; Dong et al. 2011), drought (Wilkins et al. 2010), and UVB (Fehér et al. 2011; Horak and Farré 2015). Gating effects have also been described for many biotic stresses including downy mildew (Wang et al. 2011), herbivory (Goodspeed et al. 2012), *Pseudomonas* species (Bhardwaj et al. 2011; Zhang et al. 2013; Zhou et al. 2015), and *Botrytis cinerea* (Ingle et al. 2015). In *Arabidopsis*, growth (Nozue et al. 2007) and response to hormones (Covington et al. 2008) are also gated by the circadian clock. One implication of this gating is that the time of day an experiment is performed can bias the genes identified as stress-responsive. Here we examined only two time points, just after dawn and just after dusk. However, the majority of the transcriptome shows a rhythmic pattern of expression throughout the 24 h cycle (Michael et al. 2008). As a consequence, the starting transcriptional landscape could be in very different states depending on when the stress is perceived and additional variation could be found by applying heat stress at other times of day.

Ultimately, our results indicate that the time of day heat shock is applied drives variability in the physiological and transcriptomic responses in response to heat shock. Some of the genes we detected have not been previously described as heat-responsive because few experiments have been performed on plants at night. It remains to be seen if the novel heat-responsive genes actually contribute to thermotolerance, but they can serve as reporters to understand the transcriptional regulation of heat stress responses and how this is affected by the time of day. We anticipate that this temporal variation could be used to identify connections between the changes in transcriptional responses and the changes in physiology throughout the day. Identifying how these variations in response connect to the observed improvement in thermotolerance in the PM time point may provide additional pathways to improve plant tolerance to heat stress. In this study, we examined the transcriptional response after 1 h of heat shock, leaving the potential kinetic differences of the response unexplored. Transcripts we identified as not responding at one time of day may actually respond at that time, but the response may happen more quickly or slowly and may not be detectable after 1 h of heat shock. The temporal variation we observe and potential kinetic differences in transcriptional response to the same stress will facilitate elucidating comprehensive regulatory networks in the transcriptional response to heat shock. Moreover, using time as a perturbagen is a strategy that can be applied to other stresses to understand the underlying wiring of the response and how it interacts with the endogenous rhythmic patterns.

## Materials and methods

### Plant growth conditions

*Arabidopsis thaliana* (ecotype Col-0) were suspended in a 0.06% (w/v) agar solution and plated on ½ Murashige-Skoog (0.6% w/v agar) growth medium. Seedlings were grown in 12-h light/dark cycles at constant 23 °C. Experiments were performed after appearance of second set of true leaves (12 or 14-day old seedlings).

### Heat shock survival experiments

Plates of 12-day old seedlings were wrapped in aluminum foil (groups of 3 replicates) and mock treated or subjected to high heat stress (40–50 °C) 1 h after dawn (ZT 1), midday (ZT 7), dusk (ZT13), or midnight (ZT19). During the recovery period after the heat shock event, plants stressed in the AM will be in the light while PM stressed plants will be in the dark. We controlled for this variability by keeping all plates in the foil for 24 h after heat shock. For acquired thermotolerance treatments, plants were pretreated at 38 °C for 1 h in foil, then allowed to recover at 23 °C for 1 h before heat stress to simulate heat acclimation. After the heat shock treatment, plants were returned to 23 °C, 12 h light/dark cycles in foil. The foil cover was removed 24 h after each heat stress to allow for recovery. Plates of seedlings were imaged after 1, 2, 3, 4, and 5 days and returned to growth conditions. We quantified survival by recording the maximum temperature at which 50% of the plants on a plate regrow aerial tissue after the three day period. Each experiment contained a minimum of three plates per treatment. Each replicate experiment was independently performed on different days and conducted in two different chambers. To perform survival experiments in free-running conditions the plants were grown as described above until 10 days after germination. Free-running conditions were established by switching to constant light for 2 days. Heat stress was performed as described above after 2 days in constant light.

### RNA isolation and RNA-seq

Fourteen-day old seedlings were either mock treated or heat shocked. Heat stressed plants were exposed to 30 °C for 1 h at 30 min after dawn (ZT 0.5) or 30 min after dusk (ZT 12.5). After 1 h, aerial tissue was harvested and frozen in liquid nitrogen prior to RNA extraction. Purified total RNA was extracted from 4 replicates of mock treated and heat shocked plants using Qiagen RNeasy Plant Minikits, following manufacturer protocols. Samples were diluted in order to acquire two micrograms of RNA in preparation for mRNA isolation. mRNA was separated using the NEBNext Poly(A) Magnetic mRNA isolation kit (New England Biolabs). Before library preparation, mRNA samples were heated to 95 °C for 15 min to achieve 150–200 base pair fragment sizes. NEBNext Ultra RNA Library Prep Kit for Illumina (New England Biolabs) was used to generate directional libraries for sequencing. cDNA was synthesized with random hexamers. After performing end repair and adaptor ligation steps, Agencourt AMPure XP beads (Beckman Coulter) were used following recommended ratios in order to isolate 150–200 bp fragments and remove adaptors. After end repair and adaptor ligation, size selection was performed with AMPure beads to isolate 150–200 bp fragments. PCR library enrichment was performed using fifteen cycles. The libraries were run on Agilent Bioanalyzer high sensitivity DNA chip after a 1:4 (or 1:10 if the concentration is too high) dilution. NEBNext Library Quant Kit for Illumina was used to measure library quantity and quality. The libraries were diluted to 10 nmol/μl concentrations before sequencing. Sequencing was done at North Carolina State University’s Genomics Science Laboratory. The final prepared libraries were analyzed using the Illumina HiSeq 2000. Reads were then aligned to the *Arabidopsis thaliana* genome (TAIR10) using Tophat2 (v2.1.0) (Kim et al. 2013). Counts were obtained using htseq-count (v0.6.0) (Anders et al. 2015); non-default parameters included reverse strand alignment and intersection not empty. The total genes after htseq-count was 33,601. We filtered unreliably detected transcripts by retaining only genes with at least 10 counts in one sample (Online Resource 1 Fig. S7). This resulted in a total of 21,339 genes for differential expression analysis. All data have been uploaded to GSE119330 and are available for visualization at http:/go.ncsu.edu/clockworkviridi.

### Differential expression analysis 1

To generate the differential expression data for the standard categorization, read count data was split into two tables: one containing AM control and treatment data, the second containing PM control and treatment data. Read counts were analyzed for differential expression using DeSeq 2.0 at each time of day independently to profile differentially expressed transcripts in response to treatment (Love et al. 2014). Linear model design structure was based on two groups: design = ~ condition (untreated vs. treated). All analysis methods are available at https://github.com/DohertyLab/TimeOfDay-Transcriptional-Analysis.

### Differential expression analysis 2

To generate differential expression data for the refined categorization, read count data was analyzed as a whole, considering sample variation across all control and treatment data simultaneously. Read counts were analyzed for differential expression using DeSeq 2.0 with linear model design structure based on four groups: design = ~ group (AM control, AM heat, PM control, PM heat). Differentially expressed transcripts were acquired by comparing AM control to AM heat and PM control to PM heat. We considered genes to have different basal levels if the fold change between AM control and PM control > 0.5. Differentially expressed genes were called if the measure of significance was *p* value (adjusted) < 0.05 and fold change > 0.5. For this analysis, absolute levels of gene expression were not considered during the classification of genes into the 18 categories.

### Differential expression analysis 3

To generate differential expression data for our top time of day dependent candidates, read count data was analyzed as a whole, considering sample variation across all control and treatment data simultaneously. Read counts were analyzed for differential expression using DeSeq 2.0 with linear model design structure which considers the interaction of the effects of time and temperature: design = ~ Condition + Time + Condition:Time (treatment, time of day, interaction of treatment and time of day). The heatmap of this data (Fig. 5) was generated using R package “pheatmap” with parameters (scale = “row”, cuttree_rows = 5) using raw count data of the genes from interaction term differential expression analysis.

### Gene ontology (GO) analysis

Functional enrichment for the combined network (Fig. 4 and Online Resource 1 Fig. S6) were constructed using the BiNGO plugin for Cytoscape (Species alignment: Arabidopsis, Significance level < 0.05) (Maere et al. 2005). GOSlim was used for down-regulated genes for visualization simplicity. Gene ontology analysis for the standard categorization (Online Resource 3) and each category of the refined categorization (Online Resource 5) was performed using the online web tool Panther Gene List Analysis (www.pantherdb.org) (Thomas et al. 2003; Mi et al. 2017). Gene lists were analyzed using the following parameters (Statistical overrepresentation test, Reference List = Arabidopsis thaliana (all genes in database), Annotation Data Set = GO biological process complete, Test Type = Fisher’s Exact with FDR multiple test correction).

### qRT-PCR and RNA-Seq analysis in constant light

Plants grown as described above for 12 days were switched to constant light. After 2 days, the 14 day old seedlings were either mock treated or heat stressed. Heat stressed plants were exposed to 30 °C for 1 h at 30 min after dawn (ZT 0.5) or 30 min after dusk (ZT 12.5). After 1 h, aerial tissue was harvested and frozen in liquid nitrogen prior to RNA extraction. RNA extraction, sequencing library preparation, sequencing, alignment, and analysis were performed as described above, except only one replicate was sequenced. With only one replicate we were not able to statistically evaluate the responses of the samples in LL genome-wide. Therefore, to obtain an estimate of the conservation between the response of the plants in continuous light to the four replicates in constant light conditions, we compared the correlation using Pearson’s correlation. Transcript with a correlation greater than 0.7 Pearson’s correlation coefficient clearly showed a similar pattern, we set a threshold of above 0.7 as strongly correlated and classified these as having a similar response in continuous light. Transcripts between 0.5 and 0.7 were manually evaluated. Transcript responses were estimated to be conserved if the pattern of response in continuous light showed the same pattern as in diel conditions. Validation of two genes was performed on four replicates by qRT-PCR. cDNA for each sample was synthesized from 500 ng of total RNA using the Bio-Rad iScript Reverse Transcription Supermix for RT-qPCR following manufacturer recommended instructions. Expression levels of samples were normalized to the housekeeping genes IPP2 and Syntaxin (SYP61). Primers for all genes are in supplemental information (Online Resource 9). Data analysis was performed using the Bio-Rad CFX Software following the calculation methods described in (Pfaffl 2001; Vandesompele et al. 2002). Expression data is reported in terms of ΔΔCq and normalized to control PM levels.

### Enrichment calculations for comparisons to other datasets

To calculate enrichment of cycling genes in our dataset, we compared the fraction of genes cycling in our dataset (determined by having different basal levels) to the fraction of genes cycling in other datasets (determined by cycling calls made in their analysis) using hypergeometric enrichment (Michael et al. 2008). For comparisons made against the heat shock experiments by Shedge et al. (2010) and Nguyen et al. (2014), we identified differentially expressed genes based on the significance and fold change cutoffs we used (*p* value < 0.05, Fold change > 0.5), and removed genes that were either not on the microarray or did not pass the detection threshold in our samples from all calculations. A summary of these calculations can be found in the supplemental data (Online Resource 8).

## Electronic supplementary material

Below is the link to the electronic supplementary material.

Supplementary material 1 (DOCX 408041 kb)

Supplementary material 2 (XLSX 723 kb)

Supplementary material 3 (XLSX 290 kb)

Supplementary material 4 (XLSX 607 kb)

Supplementary material 5 (XLSX 29 kb)

Supplementary material 6 (XLSX 267 kb)

Supplementary material 7 (XLSX 56 kb)

Supplementary material 8 (XLSX 12 kb)

Supplementary material 9 (XLSX 11 kb)
