# Supplementary material for: Novel transcriptional responses to heat revealed by turning up the heat at night
Source: Plant Mol Biol. 2019 May 6;101(1):1–19. doi: 10.1007/s11103-019-00873-3 (PMC6695350; doi:10.1007/s11103-019-00873-3)
Supplement: Supplementary file 1 — Supplementary material 1 (DOCX 408041 kb) [file 11103_2019_873_MOESM1_ESM.docx]

**Novel transcriptional responses to heat revealed by turning up the heat at night**

*Plant Molecular Biology*

Grinevich, Dmitry O.^1^, Desai, Jigar S.^1^, Stroup, Kevin P.^1^, Duan, Jiaqi^1^, Slabaugh, Erin^1^, Doherty, Colleen J.^1^

Affiliation:

^1^Department of Molecular and Structural Biochemistry, North Carolina State University.

[cjdohert@ncsu.edu](mailto:cjdohert@ncsu.edu)

**ESM 1 Electronic Supplementary Material Figures S1-S5, Supplementary Methods, and ESM captions**

Supplemental Figures

**ESM Fig. S1 Time of day influences heat stress tolerance in both acclimated and non-acclimated Arabidopsis seedlings under circadian conditions.** Results from one experiment of *Arabidopsis* seedling heat tolerance after 1h acclimation at 37°C across four times of day (1h after Dawn (AM) - ZT 1, Midday - ZT 7, 1h after Dusk (PM) - ZT 13, Midnight - ZT 19) performed on the second day after transfer to constant light. Each black square is six individual plates, three acclimated and three non-acclimated for each treatment temperature. Images were taken of all plates as a single image. The image was cropped around the plates in groups of two, three, and six to compress the image to form this figure.

**ESM Fig. S2 Heat stress associated genes show temporal variation in unstressed conditions.** Phase distribution of heat stress regulators across morning (yellow), midday (orange), and night (blue). The phase where the peak expression occurs is indicated for transcripts with cycling MBPMA values ≥ 0.8, determined by (Mockler et al. 2008). MBPMA values are based on the model-based pattern matching algorithm developed to evaluate the correlation between patterns of gene expression and user-defined models, in this reference to identify cycling genes (Michael et al. 2008).

**1,2 -** (Miller and Mittler 2006; Hwang et al. 2014)

**3,4,5 -** (Sakuma et al. 2006; Schramm et al. 2008; Yoshida et al. 2008)

**6,7 -** (Fortunati et al. 2008; Liu et al. 2013)

**8 -** (Prasad et al. 2010)

**9,10,11,12,13,14,15,16 -** (Hobel et al. 1994; Mishra et al. 2002; Wunderlich et al. 2003; Lohmann et al. 2004; Yoshida et al. 2011; Saidi et al. 2011; Liu and Charng 2012; Guan et al. 2013)

**13,15,16 -** (Saidi et al. 2011; Liu and Charng 2012; Guan et al. 2013)

**18,19,20 -** (Davletova et al. 2005; Scarpeci et al. 2008; Perez-Salamo et al. 2014)

**21,12,13,14,15,16,22 -** (Prändl et al. 1998; Lohmann et al. 2004; Yoshida et al. 2011; Saidi et al. 2011; Liu and Charng 2012; Guan et al. 2013; Guan et al. 2014)

**1,23 -** (Miller and Mittler 2006; Giesguth et al. 2015)

**24 -** (Baniwal et al. 2006)

**25,22 -** (Nishizawa et al. 2006; Guan et al. 2014)

**3,4,5,26,14,16,27 -** (Sakuma et al. 2006; Schramm et al. 2008; Yoshida et al. 2008; Chen et al. 2010; Saidi et al. 2011; Guan et al. 2013; Song et al. 2016)

**25,29,30,19,32,34,35,33,14,7 -** (Nishizawa et al. 2006; Charng et al. 2007; Ogawa et al. 2007; Scarpeci et al. 2008; Nishizawa-Yokoi et al. 2009; Banti et al. 2010; Cohen-Peer et al. 2010; Nishizawa-Yokoi et al. 2011; Saidi et al. 2011; Liu et al. 2013)

**22 -** (Guan et al. 2014)

**37 -** (Li et al. 2010)

**38,39 -** (Panchuk et al. 2002; Li et al. 2005)

**41,42,43 -** (Song et al. 2009; Song et al. 2010; Chong et al. 2015)

**13,15,16 -** (Yoshida et al. 2011; Liu and Charng 2012; Guan et al. 2013)

**44,45 -** (Rizhsky et al. 2004; Palmblad et al. 2008)

**46 -** (Gao et al. 2008)

**22 -** (Guan et al. 2014)

**48 -** (Liu et al. 2008)

**49,50,51,52,53,54,55,56,57,58,59 -** (Queitsch et al. 2000; Hong and Vierling 2000; Agarwal et al. 2003; Katiyar-Agarwal et al. 2003; Zhang and Guy 2005; Larkindale et al. 2005; Tonsor et al. 2008; Kim et al. 2012; Zupanska et al. 2013; Wu et al. 2013; Merret et al. 2017)

**61,62 -** (Ikeda et al. 2011; Kolmos et al. 2014)

**63,64,65,66 -** (Suzuki et al. 2005; Suzuki et al. 2008; Pamela Arce et al. 2010; Suzuki et al. 2011)

**67,68,69 -** (Aviezer-Hagai et al. 2007; Meiri and Breiman 2009; Meiri et al. 2010)

**33 -** (Nishizawa-Yokoi et al. 2011)

**25,39,70,71,72 -** (Panchuk et al. 2002; Nishizawa et al. 2006; Suzuki et al. 2013; Jung et al. 2013; Wang et al. 2016)

**75 -** (Larkindale and Vierling 2007)

**73,74 -** (Kotak et al. 2007; Babu et al. 2008)

**ESM Fig. S3 Phase Distribution of Heat Stress Response Genes and Regulators Across Different Times of Day. a** Evaluation of rhythmicity of *Arabidopsis* heat response regulators (identified from published data) or heat response genes (GO category “Response to Heat”).  Enrichment of transcripts in each category with model-based pattern matching algorithm (MBPMA) score ≥ 0.8 across two diel (Smith et al. 2004; Bläsing et al. 2005) and two circadian (Harmer et al. 2000; Edwards et al. 2006) data sets. Dotted red line represents statistical significance of *p* value <0.05. **b** Circadian phase enrichment of heat stress response genes across specific phases. Significance levels are represented by *p* value < 0.05 (dashed line) and *p* value < 0.01 (solid line) across two diel data sets.

**ESM Fig. S4 Expression levels of Heat Stress Response Regulators (HRRs) and Temporally Response Genes in the AM and PM time points.** Normalized expression levels of heat stress response regulators (HRRs): HSFA2, MBF1c, HSFB2A, and temporally responsive genes: HSP26.5, HSP90.1, HSP70, HSFA7A, and APX2. HSFA class transcription factors HSFA1A, HSFA1D, HSFA1E, HSFA1B, and molecular chaperone HSP21; other heat response network genes DREB2A, HSP90.2, HSFA7B, and HSP101; PM specific heat response genes DREB2C and HSFA3; MBF1c regulon genes AT2G27580 (AN1-like), AT2G44130 (Kelch repeat F-box protein), and AT3G13310 (DJC66); genes involved in heat stress memory ROF1, HSP22, HSP18.2, and HSA32; Phytochrome Interacting Factors PIF1 and PIF4; YUC8, an auxin biosynthetic gene; and TOC1 a circadian clock component. Time points are AM control (Light Red), AM heat shock (Dark Red), PM control (Light Blue), and PM heat shock (Dark Blue). Genes in this figure are presented in the order in which they appear in the text (left to right, top to bottom).

**ESM Fig. S5 Differential Expression Response of PIF5. a** Counts per million (CPM) plotted for each of the 4 replicates of PIF5 collected at each time of day and both treatments. **b** Fold change plotted at each time point. AM fold change is calculated as AM control versus AM treated, and PM fold change is calculated as PM control versus PM treated. The CPM data displayed here are the same data which are displayed for PIF5 expression in Figure 3.

**ESM Fig. S6 Functional categorization of the downregulated transcripts in the AM and PM time points.** Gene functional networks were constructed for genes downregulated in the AM, PM, and both times of day. This analysis was performed using GOslim to focus the resulting GO terms into general categories. **a** Functional enrichment network of genes downregulated both times of day (purple), including categories such as cell death, general stress response, and lipid metabolic processes. Significance levels are designated by shading of each color. **b** Functional enrichment network of genes downregulated only in the PM (blue), including categories such as DNA metabolic processes and cell cycle. **c** Functional enrichment network of genes downregulated uniquely in the AM (red), including categories such as response to stimulus, protein modification processes, and carbohydrate metabolic processes.

**ESM Fig. S7 Selection of Cutoff for Reliably Detected Transcripts.** Histograms of log_2_ transformed average counts for each gene in the dataset. Left panel shows the unfiltered data, with a high frequency of genes with values below 0. Right panel shows the same data after filtering by removing genes which do not have at least ten counts in at least one sample. Therefore, we used at least ten counts in one sample as a cutoff for reliably detected transcripts.

Supplemental Experimental Procedures

- **Cycling Enrichment and Phase Enrichment**

The group of 184 heat shock responsive genes was identified by using loci from gene ontology category “Response to Heat” (GO:0009408). The group of 39 heat shock regulators was identified through literature review, including known regulatory genes directly involved in the *Arabidopsis* heat shock response pathway. Two heat shock regulators were not identified on the array and were therefore excluded from analysis. Thirty of these regulators showed rhythmic expression in the datasets analyzed. We determined if genes were cycling using the Diurnal tool (diurnal.mocklerlab.org). Previously, a cutoff of model-based, pattern-matching algorithm (MBPMA) was developed to identify cycling genes; MBPMA values are based on the model-based pattern matching algorithm developed to evaluate the correlation between patterns of gene expression and user-defined models, in this reference to identify cycling genes (Mockler et al. 2008). We considered MBPMA values > 0.8 as strongly rhythmic. We tested for enrichment of cycling using hypergeometric enrichment, comparing the number of cycling genes in each group of regulators or responders against the total number of cycling genes detected on the ATH1 array. Peak expression phases of heat shock regulators were determined from the Diurnal data set. Enrichment for specific phases of expression among the heat shock responders and regulators was calculated using the Phaser tool (phaser.mocklerlab.org).

- Evaluation of Basal Expression of Heat Stress Genes

We examined the daily transcriptional patterns of several known regulators of heat stress to determine if their expression in unstressed conditions was consistent throughout the day or showed a rhythmic expression pattern. The heat response regulators (**HRR)** detected on the ATH1 microarray were enriched for transcripts with a rhythmic pattern of expression (≥0.8 MBPMA) in two diel time courses and neither of the two circadian time courses (Harmer et al. 2000; Smith et al. 2004; Bläsing et al. 2005; Edwards et al. 2006; Mockler et al. 2008) (Fig. S3a). Observing that many HRRs showed a rhythmic expression pattern, we evaluated when the peak of expression occurred to determine if there was a time of day when most of these HRRs peaked in expression. The peak of expression of the 30 rhythmically expressed regulators was not cohesively regulated as a group but was distributed widely throughout both the day and night periods (Fig. S2). We observed this distribution of expression in both the diel and circadian experiments. We also examined cycling enrichment for the heat responsive genes (**HRG**) using loci from gene ontology category “Response to Heat” (GO:0009408). We tested cycling enrichment for the HRGs in two diel and two circadian datasets and found no enrichment across all four of these datasets. We examined the peak phase of expression of the rhythmic subset of HRGs and evaluated if the expression of these transcripts is restricted to any specific time of day (Fig. S2b). In both diel datasets, more HRGs peak in expression at 8h after dawn, than would be expected given the background of the data sets (*p* value < 0.01). Time points ZT 4, and 13 are also enriched in diel time series 1 and ZT11 in diel time series 2 (*p* value < 0.05).

**ESM_2 (Separate file) Differentially Expressed Genes in AM, PM, and Both.** Gene lists from standard categorization, analysis 1 (Fig. 2). Each DE identified transcript and the associated average of normalized count values divided by size factors taken from all samples across the dataset (baseMean), log_2_ based fold change between heat shock and control at that time point (log2FoldChange), the standard error estimate for the log_2_ fold change estimate (lfcSE), Wald statistic (stat), significance score of the differential expression (pvalue), Benjamini-Hochberg adjusted *p* value (padj).

**ESM_3 (Separate file) Gene Ontology Analysis Data of Standard Categorization.** Enrichment analysis for Gene Ontology terms using PANTHER for the standard categorization (Fig. 2). For each enriched GO term, the columns represent; the number of genes in the genome (Arabidopsis thaliana REFLIST), the number in the DE set indicated, the expected number in each GO term, if it is over or underrepresented, the fold enrichment, and the Bonferroni corrected significance (*p* value).

**ESM_4 (Separate file) Differentially Expressed Transcripts Grouped by Refined Categorization.** Gene lists indicating which transcripts are in each category identified by Analysis 2 (Fig. 3) Each DE identified transcript and the associated average of normalized count values divided by size factors taken from all samples across the dataset (baseMean), log_2_ based fold change between heat shock and control at that time point (log2FoldChange), the standard error estimate for the log_2_ fold change estimate (lfcSE), Wald statistic (stat), significance score of the differential expression (pvalue), Benjamini-Hochberg adjusted *p* value (padj).

**ESM_5 (Separate file) Gene Ontology Analysis of Refined Categorization.** All enriched PANTHER identified GO terms (*p* value < 0.05) in the refined categorization are listed. The significance score in each category from the refined categorization is shown.

**ESM_6 (Separate file) Promoter Analysis of Refined Categorization.** *Cis-*regulatory elements identified as enriched in each of the potential regulatory clusters (Fig. 3) using HOMER2 (Heinz et al. 2010).

**ESM_7 (Separate file) Time of Day Dependent Transcript Groups.** The 572 transcripts identified as time of day dependent (Fig. 7). Each transcript is listed with the group it clustered with and the normalized counts indicating the expression levels in the AM and PM control and heat shock time points.

**ESM_8 (Separate file) Enrichment Calculations for Comparison to Other Datasets.** Comparisons and values used to calculate hypergeometric enrichment scores for time-independent and time-specific genes found in our dataset versus heat-responsive transcripts identified in previous datasets.

**ESM_9 (Separate file) Primers Used for qRT-PCR.** Primer sequences for constant light gene expression analysis by qRT-PCR.

SI References

Agarwal M, Sahi C, Katiyar-Agarwal S, Agarwal S, Young T, Gallie DR, Sharma VM, Ganesan K, Grover A (2003) Molecular characterization of rice hsp101: complementation of yeast hsp104 mutation by disaggregation of protein granules and differential expression in indica and japonica rice types. Plant Mol Biol 51:543–53

Aviezer-Hagai K, Skovorodnikova J, Galigniana M, Farchi-Pisanty O, Maayan E, Bocovza S, Efrat Y, von Koskull-Döring P, Ohad N, Breiman A (2007) Arabidopsis immunophilins ROF1 (AtFKBP62) and ROF2 (AtFKBP65) exhibit tissue specificity, are heat-stress induced, and bind HSP90. Plant Mol Biol 63:237–255 . doi: 10.1007/s11103-006-9085-z

Babu M, Griffiths JS, Huang T-S, Wang A (2008) Altered gene expression changes in Arabidopsis leaf tissues and protoplasts in response to Plum pox virus infection. BMC Genomics 9:325 . doi: 10.1186/1471-2164-9-325

Baniwal SK, Chan KY, Scharf K-D, Nover L (2006) Role of Heat Stress Transcription Factor HsfA5 as Specific Repressor of HsfA4. J Biol Chem 282:3605–3613 . doi: 10.1074/jbc.M609545200

Banti V, Mafessoni F, Loreti E, Alpi A, Perata P (2010) The Heat-Inducible Transcription Factor HsfA2 Enhances Anoxia Tolerance in Arabidopsis. PLANT Physiol 152:1471–1483 . doi: 10.1104/pp.109.149815

Bläsing OE, Gibon Y, Günther M, Höhne M, Morcuende R, Osuna D, Thimm O, Usadel B, Scheible W-R, Stitt M (2005) Sugars and Circadian Regulation Make Major Contributions to the Global Regulation of Diurnal Gene Expression in Arabidopsis. Plant Cell Online 17:3257–3281

Charng Y, Liu H, Liu N, Chi W, Wang C, Chang S, Wang T (2007) A Heat-Inducible Transcription Factor, HsfA2, Is Required for Extension of Acquired Thermotolerance in Arabidopsis. Plant Physiol 143:251 . doi: 10.1104/PP.106.091322

Chen H, Hwang JE, Lim CJ, Kim DY, Lee SY, Lim CO (2010) Arabidopsis DREB2C functions as a transcriptional activator of HsfA3 during the heat stress response. Biochem Biophys Res Commun 401:238–244 . doi: 10.1016/j.bbrc.2010.09.038

Chong LP, Wang Y, Gad N, Anderson N, Shah B, Zhao R (2015) A highly charged region in the middle domain of plant endoplasmic reticulum (ER)-localized heat-shock protein 90 is required for resistance to tunicamycin or high calcium-induced ER stresses. J Exp Bot 66:113–124 . doi: 10.1093/jxb/eru403

Cohen-Peer R, Schuster S, Meiri D, Breiman A, Avni A (2010) Sumoylation of Arabidopsis heat shock factor A2 (HsfA2) modifies its activity during acquired thermotholerance. Plant Mol Biol 74:33–45 . doi: 10.1007/s11103-010-9652-1

Davletova S, Schlauch K, Coutu J, Mittler R (2005) The zinc-finger protein Zat12 plays a central role in reactive oxygen and abiotic stress signaling in Arabidopsis. Plant Physiol 139:847–56 . doi: 10.1104/pp.105.068254

Edwards KD, Anderson PE, Hall A, Salathia NS, Locke JCW, Lynn JR, Straume M, Smith JQ, Millar AJ (2006) FLOWERING LOCUS C Mediates Natural Variation in the High-Temperature Response of the Arabidopsis Circadian Clock. Plant Cell Online 18:639–650

Fortunati A, Piconese S, Tassone P, Ferrari S, Migliaccio F (2008) A new mutant of Arabidopsis disturbed in its roots, right-handed slanting, and gravitropism defines a gene that encodes a heat-shock factor. J Exp Bot 59:1363–1374 . doi: 10.1093/jxb/ern047

Gao H, Brandizzi F, Benning C, Larkin RM (2008) A membrane-tethered transcription factor defines a branch of the heat stress response in Arabidopsis thaliana. Proc Natl Acad Sci 105:16398–16403 . doi: 10.1073/pnas.0808463105

Giesguth M, Sahm A, Simon S, Dietz K-J (2015) Redox-dependent translocation of the heat shock transcription factor AtHSFA8 from the cytosol to the nucleus in Arabidopsis thaliana. FEBS Lett 589:718–725 . doi: 10.1016/j.febslet.2015.01.039

Guan Q, Wen C, Zeng H, Zhu J (2013) A KH Domain-Containing Putative RNA-Binding Protein Is Critical for Heat Stress-Responsive Gene Regulation and Thermotolerance in Arabidopsis. Mol Plant 6:386–395 . doi: 10.1093/mp/sss119

Guan Q, Yue X, Zeng H, Zhu J (2014) The Protein Phosphatase RCF2 and Its Interacting Partner NAC019 Are Critical for Heat Stress-Responsive Gene Regulation and Thermotolerance in Arabidopsis. Plant Cell 26:438–453 . doi: 10.1105/tpc.113.118927

Harmer SL, Hogenesch JB, Straume M, Chang HS, Han B, Zhu T, Wang X, Kreps J a, Kay S a (2000) Orchestrated transcription of key pathways in Arabidopsis by the circadian clock. Science 290:2110–2113 . doi: 10.1126/science.290.5499.2110

Heinz S, Benner C, Spann N, Bertolino E, Lin YC, Laslo P, Cheng JX, Murre C, Singh H, Glass CK (2010) Simple Combinations of Lineage-Determining Transcription Factors Prime cis-Regulatory Elements Required for Macrophage and B Cell Identities. Mol Cell 38:576–589 . doi: 10.1016/j.molcel.2010.05.004

Hobel A, Schoffl F, Tfibingen U, Institut B, Genetik A, Morgenstelle A Der, Tiibingen D- (1994) Arabidopsis heat shock factor : isolation and characterization of the gene and the recombinant protein + ). 353–362

Hong SW, Vierling E (2000) Mutants of Arabidopsis thaliana defective in the acquisition of tolerance to high temperature stress. Proc Natl Acad Sci U S A 97:4392–7

Hwang SM, Kim DW, Woo MS, Jeong HS, Son YS, Akhther S, Choi GJ, Bahk JD (2014) Functional characterization of Arabidopsis HsfA6a as a heat-shock transcription factor under high salinity and dehydration conditions. Plant Cell Environ 37:1202–1222 . doi: 10.1111/pce.12228

Ikeda M, Mitsuda N, Ohme-Takagi M (2011) Arabidopsis HsfB1 and HsfB2b act as repressors of the expression of heat-inducible Hsfs but positively regulate the acquired thermotolerance. Plant Physiol 157:1243–54 . doi: 10.1104/pp.111.179036

Jung H-S, Crisp PA, Estavillo GM, Cole B, Hong F, Mockler TC, Pogson BJ, Chory J (2013) Subset of heat-shock transcription factors required for the early response of Arabidopsis to excess light. Proc Natl Acad Sci 110:14474–14479 . doi: 10.1073/pnas.1311632110

Katiyar-Agarwal S, Agarwal M, Grover A (2003) Heat-tolerant basmati rice engineered by over-expression of hsp101. Plant Mol Biol 51:677–86

Kim M, Lee U, Small I, des Francs-Small CC, Vierling E (2012) Mutations in an Arabidopsis Mitochondrial Transcription Termination Factor-Related Protein Enhance Thermotolerance in the Absence of the Major Molecular Chaperone HSP101. Plant Cell 24:3349–3365 . doi: 10.1105/tpc.112.101006

Kolmos E, Chow BY, Pruneda-Paz JL, Kay SA (2014) HsfB2b-mediated repression of PRR7 directs abiotic stress responses of the circadian clock. Proc Natl Acad Sci U S A 111:16172–7 . doi: 10.1073/pnas.1418483111

Kotak S, Vierling E, Baumlein H, Koskull-Doring P v. (2007) A Novel Transcriptional Cascade Regulating Expression of Heat Stress Proteins during Seed Development of Arabidopsis. PLANT CELL ONLINE 19:182–195 . doi: 10.1105/tpc.106.048165

Larkindale J, Hall JD, Knight MR, Vierling E (2005) Heat Stress Phenotypes of Arabidopsis Mutants Implicate Multiple Signaling Pathways in the Acquisition of Thermotolerance. PLANT Physiol 138:882–897 . doi: 10.1104/pp.105.062257

Larkindale J, Vierling E (2007) Core Genome Responses Involved in Acclimation to High Temperature. Plant Physiol 146:748–761 . doi: 10.1104/pp.107.112060

Li C, Chen Q, Gao X, Qi B, Chen N, Xu S, Chen J, Wang X (2005) AtHsfA2 modulates expression of stress responsive genes and enhances tolerance to heat and oxidative stress in Arabidopsis. Sci China Ser C, Life Sci 48:540–50

Li S, Zhou X, Chen L, Huang W, Yu D (2010) Functional characterization of Arabidopsis thaliana WRKY39 in heat stress. Mol Cells 29:475–483 . doi: 10.1007/s10059-010-0059-2

Liu H-T, Gao F, Li G-L, Han J-L, Liu D-L, Sun D-Y, Zhou R-G (2008) The calmodulin-binding protein kinase 3 is part of heat-shock signal transduction in *Arabidopsis thaliana*. Plant J 55:760–773 . doi: 10.1111/j.1365-313X.2008.03544.x

Liu H, Charng Y (2012) Acquired thermotolerance independent of heat shock factor A1 (HsfA1), the master regulator of the heat stress response. Plant Signal Behav 7:547–550 . doi: 10.4161/psb.19803

Liu J, Sun N, Liu M, Liu J, Du B, Wang X, Qi X (2013) An Autoregulatory Loop Controlling Arabidopsis HsfA2 Expression: Role of Heat Shock-Induced Alternative Splicing. PLANT Physiol 162:512–521 . doi: 10.1104/pp.112.205864

Lohmann C, Eggers-Schumacher G, Wunderlich M, Sch�ffl F (2004) Two different heat shock transcription factors regulate immediate early expression of stress genes in Arabidopsis. Mol Genet Genomics 271:11–21 . doi: 10.1007/s00438-003-0954-8

Maere S, Heymans K, Kuiper M (2005) BiNGO: A Cytoscape plugin to assess overrepresentation of Gene Ontology categories in Biological Networks. Bioinformatics 21:3448–3449 . doi: 10.1093/bioinformatics/bti551

Meiri D, Breiman A (2009) Arabidopsis ROF1 (FKBP62) modulates thermotolerance by interacting with HSP90.1 and affecting the accumulation of HsfA2-regulated sHSPs. Plant J 59:387–399 . doi: 10.1111/j.1365-313X.2009.03878.x

Meiri D, Tazat K, Cohen-Peer R, Farchi-Pisanty O, Aviezer-Hagai K, Avni A, Breiman A (2010) Involvement of Arabidopsis ROF2 (FKBP65) in thermotolerance. Plant Mol Biol 72:191–203 . doi: 10.1007/s11103-009-9561-3

Merret R, Carpentier M-C, Favory J-J, Picart C, Descombin J, Bousquet-Antonelli C, Tillard P, Lejay L, Deragon J-M, Charng Y (2017) Heat Shock Protein HSP101 Affects the Release of Ribosomal Protein mRNAs for Recovery after Heat Shock. Plant Physiol 174:1216–1225 . doi: 10.1104/pp.17.00269

Mi H, Huang X, Muruganujan A, Tang H, Mills C, Kang D, Thomas PD (2017) PANTHER version 11: Expanded annotation data from Gene Ontology and Reactome pathways, and data analysis tool enhancements. Nucleic Acids Res 45:D183–D189 . doi: 10.1093/nar/gkw1138

Miller G, Mittler R (2006) Could heat shock transcription factors function as hydrogen peroxide sensors in plants? Ann Bot 98:279–288 . doi: 10.1093/aob/mcl107

Mishra SK, Tripp J, Winkelhaus S, Tschiersch B, Theres K, Nover L, Scharf K-D (2002) In the complex family of heat stress transcription factors, HsfA1 has a unique role as master regulator of thermotolerance in tomato. Genes Dev 16:1555–1567 . doi: 10.1101/gad.228802

Mockler TC, Michael TP, Breton G, McEntee C, Byer A, Trout JD, Hazen SP, Shen R, Priest HD, Sullivan CM, Givan SA, Yanovsky M, Hong F, Kay SA, Chory J (2008) Network Discovery Pipeline Elucidates Conserved Time-of-Day-Specific cis-Regulatory Modules. PLoS Genet 4:

Nishizawa-Yokoi A, Nosaka R, Hayashi H, Tainaka H, Maruta T, Tamoi M, Ikeda M, Ohme-Takagi M, Yoshimura K, Yabuta Y, Shigeoka S (2011) HsfA1d and HsfA1e Involved in the Transcriptional Regulation of HsfA2 Function as Key Regulators for the Hsf Signaling Network in Response to Environmental Stress. Plant Cell Physiol 52:933–945 . doi: 10.1093/pcp/pcr045

Nishizawa-Yokoi A, Yoshida E, Yabuta Y, Shigeoka S (2009) Analysis of the Regulation of Target Genes by an Arabidopsis Heat Shock Transcription Factor, HsfA2. Biosci Biotechnol Biochem 73:890–895 . doi: 10.1271/bbb.80809

Nishizawa A, Yabuta Y, Yoshida E, Maruta T, Yoshimura K, Shigeoka S (2006) Arabidopsis heat shock transcription factor A2 as a key regulator in response to several types of environmental stress. Plant J 48:535–547 . doi: 10.1111/j.1365-313X.2006.02889.x

Ogawa D, Yamaguchi K, Nishiuchi T (2007) High-level overexpression of the Arabidopsis HsfA2 gene confers not only increased themotolerance but also salt/osmotic stress tolerance and enhanced callus growth. J Exp Bot 58:3373–3383 . doi: 10.1093/jxb/erm184

Palmblad M, Mills DJ, Bindschedler L V. (2008) Heat-Shock Response in Arabidopsis thaliana Explored by Multiplexed Quantitative Proteomics Using Differential Metabolic Labeling. J Proteome Res 7:780–785 . doi: 10.1021/pr0705340

Pamela Arce D, Verónica Godoy A, Tsuda K, Yamazaki K, Marta Valle E, José Iglesias M, Di Mauro MF, Anahí Casalongué C (2010) The analysis of an Arabidopsis triple knock-down mutant reveals functions for MBF1 genes under oxidative stress conditions. J Plant Physiol 167:194–200 . doi: 10.1016/j.jplph.2009.09.003

Panchuk II, Volkov RA, Schöffl F (2002) Heat Stress- and Heat Shock Transcription Factor-Dependent Expression and Activity of Ascorbate Peroxidase in Arabidopsis. PLANT Physiol 129:838–853 . doi: 10.1104/pp.001362

Perez-Salamo I, Papdi C, Rigo G, Zsigmond L, Vilela B, Lumbreras V, Nagy I, Horvath B, Domoki M, Darula Z, Medzihradszky K, Bogre L, Koncz C, Szabados L (2014) The Heat Shock Factor A4A Confers Salt Tolerance and Is Regulated by Oxidative Stress and the Mitogen-Activated Protein Kinases MPK3 and MPK6. PLANT Physiol 165:319–334 . doi: 10.1104/pp.114.237891

Prändl R, Hinderhofer K, Eggers-Schumacher G, Schöffl F (1998) HSF3, a new heat shock factor from Arabidopsis thaliana, derepresses the heat shock response and confers thermotolerance when overexpressed in transgenic plants. Mol Gen Genet 258:269–78

Prasad BD, Goel S, Krishna P (2010) In silico identification of carboxylate clamp type tetratricopeptide repeat proteins in Arabidopsis and rice as putative co-chaperones of Hsp90/Hsp70. PLoS One 5:e12761 . doi: 10.1371/journal.pone.0012761

Queitsch C, Hong SW, Vierling E, Lindquist S (2000) Heat shock protein 101 plays a crucial role in thermotolerance in Arabidopsis. Plant Cell 12:479–92

Rizhsky L, Liang H, Shuman J, Shulaev V, Davletova S, Mittler R (2004) When defense pathways collide. The response of Arabidopsis to a combination of drought and heat stress. Plant Physiol 134:1683–96 . doi: 10.1104/pp.103.033431

Saidi Y, Finka A, Goloubinoff P (2011) Heat perception and signalling in plants: a tortuous path to thermotolerance. New Phytol 190:556–565 . doi: 10.1111/j.1469-8137.2010.03571.x

Sakuma Y, Maruyama K, Osakabe Y, Qin F, Seki M, Shinozaki K, Yamaguchi-Shinozaki K (2006) Functional analysis of an Arabidopsis transcription factor, DREB2A, involved in drought-responsive gene expression. Plant Cell 18:1292–309 . doi: 10.1105/tpc.105.035881

Scarpeci TE, Zanor MI, Valle EM (2008) Investigating the role of plant heat shock proteins during oxidative stress. Plant Signal Behav 3:856–7

Schramm F, Larkindale J, Kiehlmann E, Ganguli A, Englich G, Vierling E, Von Koskull-Döring P (2008) A cascade of transcription factor DREB2A and heat stress transcription factor HsfA3 regulates the heat stress response of Arabidopsis. Plant J 53:264–274 . doi: 10.1111/j.1365-313X.2007.03334.x

Smith SM, Fulton DC, Chia T, Thorneycroft D, Chapple A, Dunstan H, Hylton C, Zeeman SC, Smith AM (2004) Diurnal Changes in the Transcriptome Encoding Enzymes of Starch Metabolism Provide Evidence for Both Transcriptional and Posttranscriptional Regulation of Starch Metabolism in Arabidopsis Leaves. Plant Physiol 136:2687–2699

Song C, Chung WS, Lim CO (2016) Overexpression of Heat Shock Factor Gene HsfA3 Increases Galactinol Levels and Oxidative Stress Tolerance in Arabidopsis. Mol Cells 39:477–83 . doi: 10.14348/molcells.2016.0027

Song H, Fan P, Shi W, Zhao R, Li Y (2010) Expression of five AtHsp90 genes in Saccharomyces cerevisiae reveals functional differences of AtHsp90s under abiotic stresses. J Plant Physiol 167:1172–1178 . doi: 10.1016/j.jplph.2010.03.016

Song H, Zhao R, Fan P, Wang X, Chen X, Li Y (2009) Overexpression of AtHsp90.2, AtHsp90.5 and AtHsp90.7 in Arabidopsis thaliana enhances plant sensitivity to salt and drought stresses. Planta 229:955–964 . doi: 10.1007/s00425-008-0886-y

Suzuki N, Bajad S, Shuman J, Shulaev V, Mittler R (2008) The Transcriptional Co-activator MBF1c Is a Key Regulator of Thermotolerance in Arabidopsis thaliana. J Biol Chem 283:9269–9275 . doi: 10.1074/jbc.M709187200

Suzuki N, Miller G, Sejima H, Harper J, Mittler R (2013) Enhanced seed production under prolonged heat stress conditions in Arabidopsis thaliana plants deficient in cytosolic ascorbate peroxidase 2. J Exp Bot 64:253–263 . doi: 10.1093/jxb/ers335

Suzuki N, Rizhsky L, Liang H, Shuman J, Shulaev V, Mittler R (2005) Enhanced tolerance to environmental stress in transgenic plants expressing the transcriptional coactivator multiprotein bridging factor 1c. Plant Physiol 139:1313–22 . doi: 10.1104/pp.105.070110

Suzuki N, Sejima H, Tam R, Schlauch K, Mittler R (2011) Identification of the MBF1 heat-response regulon of Arabidopsis thaliana. Plant J 66:844–851 . doi: 10.1111/j.1365-313X.2011.04550.x

Tonsor SJ, Scott C, Boumaza I, Liss TR, Brodsky JL, Vierling E (2008) Heat shock protein 101 effects in A. thaliana: genetic variation, fitness and pleiotropy in controlled temperature conditions. Mol Ecol 17:1614–26 . doi: 10.1111/j.1365-294X.2008.03690.x

Wang X, Huang W, Yang Z, Liu J, Huang B (2016) Transcriptional regulation of heat shock proteins and ascorbate peroxidase by CtHsfA2b from African bermudagrass conferring heat tolerance in Arabidopsis. Sci Rep 6:28021 . doi: 10.1038/srep28021

Wu T -y., Juan Y -t., Hsu Y -h., Wu S -h., Liao H -t., Fung RWM, Charng Y -y. (2013) Interplay between Heat Shock Proteins HSP101 and HSA32 Prolongs Heat Acclimation Memory Posttranscriptionally in Arabidopsis. PLANT Physiol 161:2075–2084 . doi: 10.1104/pp.112.212589

Wunderlich M, Werr W, Schöffl F (2003) Generation of dominant-negative effects on the heat shock response in Arabidopsis thaliana by transgenic expression of a chimaeric HSF1 protein fusion construct. Plant J 35:442–51

Yoshida T, Ohama N, Nakajima J, Kidokoro S, Mizoi J, Nakashima K, Maruyama K, Kim JM, Seki M, Todaka D, Osakabe Y, Sakuma Y, SchöZ F, Shinozaki K, Yamaguchi-Shinozaki K (2011) Arabidopsis HsfA1 transcription factors function as the main positive regulators in heat shock-responsive gene expression. Mol Genet Genomics 286:321–332 . doi: 10.1007/s00438-011-0647-7

Yoshida T, Sakuma Y, Todaka D, Maruyama K, Qin F, Mizoi J, Kidokoro S, Fujita Y, Shinozaki K, Yamaguchi-Shinozaki K (2008) Functional analysis of an Arabidopsis heat-shock transcription factor HsfA3 in the transcriptional cascade downstream of the DREB2A stress-regulatory system. Biochem Biophys Res Commun 368:515–521 . doi: 10.1016/j.bbrc.2008.01.134

Zhang C, Guy CL (2005) Co-immunoprecipitation of Hsp101 with cytosolic Hsc70. Plant Physiol Biochem 43:13–18 . doi: 10.1016/j.plaphy.2004.10.006

Zupanska AK, Denison FC, Ferl RJ, Paul AL (2013) Spaceflight engages heat shock protein and other molecular chaperone genes in tissue culture cells of Arabidopsis Thaliana. Am J Bot 100:235–248 . doi: 10.3732/ajb.1200343
